# CDK6 is activated by the atypical cyclin I to promote E2F‐mediated gene expression and cancer cell proliferation

**DOI:** 10.1002/1878-0261.13438

**Published:** 2023-05-31

**Authors:** Eva Quandt, Núria Masip, Sara Hernández‐Ortega, Abril Sánchez‐Botet, Laura Gasa, Ainhoa Fernández‐Elorduy, Sara Plutta, Joan Marc Martínez‐Láinez, Samuel Bru, Pau M. Munoz‐Torres, Martin Floor, Jordi Villà‐Freixa, May C. Morris, August Vidal, Alberto Villanueva, Josep Clotet, Mariana P. C. Ribeiro

**Affiliations:** ^1^ Basic Science Department, Faculty of Medicine and Health Sciences Universitat Internacional de Catalunya Barcelona Spain; ^2^ Institut de Neurociències Universitat Autònoma de Barcelona Bellaterra (Cerdanyola del Vallès) Spain; ^3^ Department of Biosciences, Faculty of Sciences, Technology and Engineering Universitat de Vic – Universitat Central de Catalunya Spain; ^4^ Institut des Biomolécules Max Mousseron, CNRS‐UMR5247 Université de Montpellier France; ^5^ Servei d'Anatomia Patològica Hospital Universitari de Bellvitge Barcelona Spain; ^6^ Oncobell Program Institut d'Investigació Biomèdica de Bellvitge (IDIBELL) Barcelona Spain; ^7^ Chemoresistance and Predictive Factors Group, Program Against Cancer Therapeutic Resistance (ProCURE) Catalan Institute of Oncology (ICO) Bellvitge Biomedical Research Institute (IDIBELL) Barcelona Spain

**Keywords:** atypical cyclins, CDK6, E2F, palbociclib, retinoblastoma

## Abstract

Cyclin‐dependent kinases (CDKs), together with their cyclin partners, are the master cell cycle regulators. Remarkably, the cyclin family was extended to include atypical cyclins, characterized by distinctive structural features, but their partner CDKs remain elusive. Here, we conducted a yeast two‐hybrid screen to identify new atypical cyclin–CDK complexes. We identified 10 new complexes, including a complex between CDK6 and cyclin I (CCNI), which was found to be active against retinoblastoma protein. *CCNI* upregulation increased the proliferation of breast cancer cells *in vitro* and *in vivo*, with a magnitude similar to that seen upon cyclin D upregulation, an effect that was abrogated by *CDK6* silencing or palbociclib treatment. In line with these findings, *CCNI* downregulation led to a decrease in cell number and a reduction in the percentage of cells reaching S phase. Finally, *CCNI* upregulation correlated with the high expression of E2F target genes in large panels of cancer cell lines and tissue samples from breast cancer patients. In conclusion, we unveil CCNI as a new player in the pathways that activate CDK6, enriching the wiring of cell cycle control.

## Graphical abstract

The authors found that the cell cycle kinase CDK6 forms an active complex with the atypical cyclin I (CCNI) and that this complex promotes retinoblastoma phosphorylation, E2F‐mediated gene expression, and tumor proliferation *in vitro* and *in vivo*, similarly to the cyclin D1 (CCND1)/CDK6 complex. This suggests that, in some contexts, CCNI and CCND1 work in parallel during G1 phase to ensure cell cycle progression.

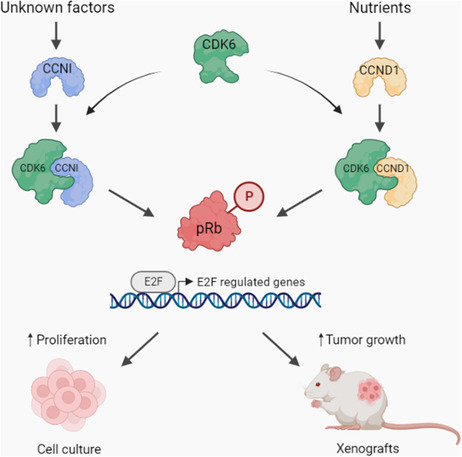

**Abbreviations**
AD: activation domain
BrdU: 5‐bromo‐2′‐deoxyuridine
CCND: D‐type cyclins
CCNI: cyclin I
CDKs: cyclin‐dependent kinases
cDNA: complementary DNA
DNA‐BD: DNA‐binding domain
EBV: Epstein–Barr virus
GSEA: Gene Set Enrichment Analysis
PNBM: paranitrobenzomesylate
pRb: retinoblastoma
SBP: structure‐based potential
siRNA: small interfering RNA

## 1 Introduction

It is well established that cyclins, through their interaction with Cyclin‐Dependent Kinases (CDKs), govern the process of cell division. The role of CDK/cyclin complexes is usually exemplified by the response of quiescent cells to extracellular mitogenic signals, when D‐type cyclins (D1, D2, and D3) are expressed and allow the heterodimerization and activation of CDK4 or CDK6. The active complexes phosphorylate and inactivate the retinoblastoma (pRb) family of proteins, leading to the derepression of E2F transcription factors and activation of transcriptional programs required for cell proliferation. Thus, pRb phosphorylation by cyclin D‐CDK4/6 is a crucial first step to promote G1‐ to S‐phase transition [1] and deregulated expression of D‐type cyclins and the resulting CDK4/6 hyperactivation is a driving force in tumorigenesis. Accordingly, the successful use of multiple CDK4/6 inhibitors in clinical practice has renewed interest in the biology of CDKs and cyclins [2].

In 2004 the Human Genome Project unveiled the existence of other proteins carrying the characteristic ‘cyclin box’ domain that determines CDK binding [3]. These new members of the cyclin family share significant similarities, a finding that led to the recent establishment of the subfamily of atypical cyclins, characterized by at least two of three defining traits: the presence of a single ‘cyclin box’, a defining Lys–Glu pair in the ‘cyclin box’ domain and a particular interactor pattern [4]. Regarding the latter, while canonical cyclins interact with members of the CDK1‐ and CDK4‐related subfamilies and transcriptional cyclins associate with transcriptional CDKs, atypical cyclins have no known interactors or interact with atypical CDKs (subfamilies of CDK5, 14, 15, 16, 17, and 18) [4, 5]. Although this distinctive feature may be explained by the particularities of the region that determines CDK binding, this assumption is limited by the lack of studies that systematically address the interactions between atypical cyclins and CDKs [4]. Interestingly, it was shown that several cell lines can proliferate in the absence of D‐type cyclins [6], suggesting that other cyclins may interact with CDKs to drive cell cycle progression. Indeed, several atypical cyclins have been shown to increase cell proliferation [7, 8, 9] and, therefore, the identification of new cyclin–CDK complexes may unveil new pathways contributing to tumor development and progression.

The identification of new interactors of atypical cyclins is not an easy task. On the one hand, the lack of crystallographic structures makes it difficult to predict new cyclin–CDK complexes *in silico*. On the other hand, high‐throughput interactomic studies were unable to reproduce the previously described complexes using this strategy [10, 11]. Nevertheless, the few described attempts to find interactions by two‐hybrid assays proved to be successful [12, 13], indicating that it might be a good strategy to detect these labile heterodimers.

In the present work, we identified new atypical cyclin–CDK complexes, such as the cyclin I (CCNI)–CDK6 complex, a finding that was confirmed through other experimental approaches. Our results show that, by interacting with CDK6, CCNI promotes the proliferation of breast cancer cell lines, unraveling a new pathway that contributes to cell cycle regulation and tumorigenesis.

## 2 Materials and methods

### 2.1 Plasmids

Constructs were prepared by recombinational cloning using the In‐FusionHD kit (Clontech, Mountain View, CA, USA). Inserts were amplified from complementary DNA (cDNA) obtained from cell lines using the cDNA synthesis kit (Bio‐Rad, Hercules, CA, USA). Cyclins and CDKs were cloned into the pGBKT7 and pGADT7 vectors included in the Matchmaker Gold Yeast Two‐Hybrid System (Clontech). To generate the CDK‐binding‐deficient CCNI mutant E103A, the following oligonucleotides were used for cloning into pGADT7: GAAGATGAGAGAATTCCAGTACTAAAGG and AATTCTCTCATCTTCAGCAACAGTCTTGGCAGCTAGG; ATGGAGGCCGAATTCGGATCCAAGTTTCCAGGGCCTTTGG and AATTCTCTCATCTTCAGCAACAGTCTTGGCAGCTAGG. To generate the CDK‐binding‐deficient CDK6 mutant R60A, the following oligonucleotides were used for cloning into pGBKT7: GAGGTGGCGGTGCTGAGG and CATCTGCAGCTCGAGGGATCCTCAGGCTGTATTCAGCTCCGAGG; GATTACGCTCATATGGGATCCGAGAAGGACGGCCTGTGCCG and CAGCACCGCCACCTCAGCGATGGTGGAGAGCGGCATG. N‐terminal GST‐tagged proteins were obtained by cloning into the pGEX6P1. For overexpression in cell lines, N‐terminal Flag‐tagged *SPY1* was cloned into the pIRES2eGFP vector. Human cDNA of *CCNI* and *CCND1* were amplified from cells with a C‐terminal Flag tag and cloned into pWPI lentiviral expression vector (#12254; Addgene, Watertown, MA, USA). To generate the CDK‐binding‐deficient CCNI mutant, the following oligonucleotides were used: Fw: GCCTCGAGGTTTAAACATGAAGTTTCCAGGGCCTTTGGAAAACCAGAGATTGTCTTTCACAGCCACACCATTGGATTTTC; Rv: (GCCCGTAGTTTAAACCTACTTATCGTCGTCATCCTTGTAATCCATGACAGAAACAGGCTG).

### 2.2 Cell line and reagents

Cells were cultured in DMEM (Sigma‐Aldrich, St. Louis, MO, USA) supplemented with 10% fetal bovine serum (Sigma‐Aldrich), 1% glutamax (Biowest, Nuaillé, France), and 1% penicillin/streptomycin (Sigma‐Aldrich), except ChaGo‐K‐1 and DU145 cells that were cultured in RPMI (Sigma‐Aldrich). All cells were grown in humidified air at 37 °C and 5% CO_2_ atmosphere. Palbociclib was purchased from Sigma‐Aldrich. Experiments were performed in mycoplasma‐free cells.

A549 (RRID:CVCL_0023), ChaGo‐K‐1 (RRID:CVCL_1121), and HT‐29 (RRID:CVCL_0320) cells were purchased from the European Collection of Authenticated Cell Cultures (ECACC). MCF7 (RRID:CVCL_0031) cells were purchased from Eucellbank (Barcelona, Spain). BT‐474 (RRID:CVCL_0179) cells were a kind gift from R. Wright. DU145 (RRID:CVCL_010) cells a kind gift from M. Olivan (Vall d'Hebron Institut de Recerca, Spain). MDA‐MB‐231 (RRID:CVCL_0062) and LoVo (RRID:CVCL_0399) cells were a kind gift from Mezquita (Universitat de Barcelona, Spain). HCA7 (RRID:CVCL_0289) cells were a kind gift from R. Mangues (Hospital Sant Pau, Barcelona). NCI‐H1437 (RRID:CVCL_1472) cells were a kind gift from M. Esteller (IDIBELL, Barcelona). All cell lines have been authenticated in the past 3 years by Short Tandem Repeat (STR) analysis. Epstein–Barr virus (EBV)‐immortalized lymphocytes were a kind gift from M. Sánchez (Universitat Internacional de Catalunya, Barcelona, Spain) and were grown in RPMI supplemented with 15% fetal bovine serum, 1% glutamax, and 1% penicillin/streptomycin.

### 2.3 Protein expression and purification

GST‐fusion proteins were expressed and purified from BL21 (DE3) *Escherichia coli* cells. Protein expression was induced with isopropyl β‐d‐thioglactopyranoside. Cell pellets were resuspended with lysis buffer (50 mm Tris–HCl pH 7.5, 150 mm NaCl, 5% Glycerol, 0.1% Triton X‐100, 1 mm EDTA, 1 mm DTT, 100 mm PMSF, 10 mg·mL^−1^ Leupeptin, 1 mg·mL^−1^ Pepstatin, 0.5 m Benzamidin). Lysozyme was added and cells were incubated for 10 min at 37 °C. STET buffer (10 mm Tris–HCl pH 8, 100 mm NaCl, 1 mm EDTA, and 5% Triton X‐100) was added, and cells were sonicated and centrifuged. The supernatants were purified using Glutathion‐Sepharose column chromatography. After incubation for 1 h at 4 °C with rotation, the beads were collected by centrifugation and washed with lysis/STET and equilibration buffers (50 m Tris–HCl pH 8, 150 mm NaCl, 1 mm MgCl_2_, and 1 mm DTT). Reduced glutathione was added for elution.

### 2.4 Yeast two‐hybrid assays

Cyclin‐dependent kinase and cyclin vectors were transformed into yeast strains Y187 mat alpha (a kind offer from R. Aligué, University of Barcelona) and AH109 mat a (Matchmaker Gold Yeast Two‐Hybrid System kit; Clontech), respectively. Both transformants were grown on ‐Trp and ‐Leu medium, respectively, at 30 °C for 48 h. One clone of each transformant was grown in the corresponding synthetic liquid medium at 30 °C for 16 h. Cyclins and CDKs were mixed (0.005 ODs of each), and plated the following day in both ‐Trp/‐Leu medium and ‐Leu/‐Trp/‐His/‐Ade selection medium. For spot‐seeding, 0.01 ODs of an exponential culture of diploid cells were serially diluted and spot‐seeded in the selection medium.

### 2.5 Protein extraction from rat brain tissue

Homogenization of brain tissue from a Sprague‐Dawley male rat (weight 300 g) at 10 weeks old, from the Animal Facility of the Faculty of Pharmacy of the University of Barcelona, was carried out in ice‐cold extraction buffer (50 mm HEPES‐KOH pH 7.4, 150 mm NaCl, 0.1% NP40 (IGEPAL CA630), 1 mm DTT) supplemented with protease and phosphatase inhibitors. The homogenate was rotated at 4 °C for 1 h and clarified by centrifugation at 18 400 *g* for 15 min at 4 °C. The amount of protein in the extract was quantified by the Bradford assay (Bio‐Rad).

The protocols for animal care and use were approved by the Clinical Research Ethics Committee of the University of Barcelona (Procedure Ref. 11113, Generalitat de Catalunya). All experimental animal procedures were carried out in strict accordance with European directive 2010/63/EU and Spanish legislation (BOE 252∕34367–91, 2005) regulating animal research. Animals were housed according to a 12 h/12 h light/dark cycle (from 8 am to 8 pm) in a temperature‐ and humidity‐controlled room and were allowed free access to water and standard laboratory chow diet.

### 2.6 Protein extraction and western blot analysis

Protein extracts from cell lines were prepared as described elsewhere [14]. Total protein was separated by 10% SDS/PAGE and transferred to PVDF membranes (Immobilon‐P; Millipore, Burlington, MA, USA). Membranes were incubated with the primary (Table S1) and secondary antibodies (Jackson Laboratories, West Grove, PA, USA). Ponceau staining (Sigma‐Aldrich) was performed to normalize protein expression.

### 2.7 Trapping assays

Protein extract (2.5–5 mg) was incubated with Glutathion‐Sepharose resin bound to GST‐fusion proteins and rotated for 16 h (or 4 h in the assays using EBV‐immortalized lymphocytes) at 4 °C, washed with lysis buffer and ultrapure water. The resin was compacted by centrifugation and transferred to a column with a 35 μm pore size filter (Mobicol, Goettingen, Germany). The resin was washed with lysis buffer supplemented with phosphatase and protease inhibitors. The resin was recovered, and a sample buffer was then added for SDS/PAGE.

### 2.8 Coimmunoprecipitation

Protein extract (3–5 mg) was incubated with α‐Flag antibody‐bound beads and rotated for 14–15 h at 4 °C. Bound proteins were washed with lysis buffer and ultrapure water. Proteins were eluted with ultrapure water and denatured with 5× SDS sample buffer. Following a 10‐min incubation at 30 °C, the proteins were separated by SDS/PAGE.

### 2.9 *In vitro* kinase assays

GST‐CDKs and GST‐Cyclins (0.4 μm) were preactivated using 0.04 μm human CAK (Sigma‐Aldrich) in a solution containing 500 mm Tris–HCl pH 7.5, 100 mm MgCl_2_, and 1 mm ATP (Sigma‐Aldrich) at 30 °C for 30 min. The samples were transferred to a 30K centrifugal filter unit (Millipore) for buffer exchange. 1 μm GST‐pRb was then added to the reaction medium containing 500 mm HEPES pH 7.5, 500 mm NaCl, 1 mm MgCl_2_, 50 mm EGTA, 10 mm DTT, and 1 mm ATP‐γ‐S (Axxora, Farmingdale, NY, USA) in the presence and absence of CDK4/6 inhibitors at 30 °C for 45 min. Reactions were quenched by adding 50 mm EDTA and incubated with 0.5 mg·mL^−1^ paranitrobenzomesylate (PNBM), kindly provided by K. M. Shokat, at 25 °C for 45 min. PNBM samples were subjected to SDS/PAGE and probed for thiophosphorylation.

### 2.10 Fluorescence kinase assays

Fluorescence kinase assays were performed using the peptide biosensor CDKACT6‐TAMRA [15]. Briefly, CDKACT6‐TAMRA (200 nm) and cell extracts (40 μg) were added to 200 μL of PBS supplemented with 5 mm MgCl_2_, 0.5 mm ATP in a 96‐well dark plate, which was placed in a microplate reader at 30 °C for 2 h. Changes in TAMRA fluorescence emission were recorded every 90 s at 570 nm following excitation at 544 nm. Relative fluorescence was calculated by subtracting CDKACT6‐TAMRA fluorescence from cell extract fluorescence.

### 2.11 Structural analysis

A search of the Protein Data Bank [16] was carried out using the ‘Molecular Function’‐go term ‘cyclin binding’ (GO ID: 30332). A set of 577 models containing cyclin structures was retrieved that hold 10 unique structures of Human CDKs bound to cyclin partners (PDB codes: 1F5Q, 1W98, 2F2C, 2JGZ, 2W96, 3G33, 3MI9, 3QHW, 4EOJ). For each complex, we defined interface residues as those having ≥ 20% of unbound Solvent Accessible Surface Area hidden upon complexation. Common interface positions present in all CDK/cyclin complexes were defined from a multiple sequence alignment carried out with the mafft program [17]. Interchain contact maps were calculated with the shadow map algorithm [18] to define the set of common CDK/cyclin interactions as all the interchain contacts found in all 10 CDK/cyclin complexes.

The comparative modeling for CCNI was constructed using the automated protocol of the i‐tasser software [19]. The sequence of the Uniprot entry of CCNI protein (ID: Q14094) was used as an input with default options. The best five predictions were energy minimized searching the local conformational space using the relax protocol of rosetta3 software [20]. A thousand minimization trajectories were produced from each i‐tasser prediction, making 5000 conformations. Only the top I‐Tasser model generated significantly low energy structures and the lowest energy one was selected as the final comparative model of CCNI.

The CCNI–CDK6 complex structures were generated using protein–protein docking calculations. The comparative model of CCNI was docked against the crystal structure of CDK6 kinase (PDB code: 2F2C) using RosettaDock local optimization protocol [21]. Interchain distances, pertaining to conserved contacts in several cyclin–CDK complexes, were constrained during the optimization to maintain the right orientation between the two chains. All distance restraints were between alpha carbon atoms and included the pairs L97‐I59, K100‐E52, K100‐G53, K100‐M54, K100‐L56, K100‐I59, T101‐I59, T101‐A63, E106‐R60, L111‐E52, E131‐E52, E131‐G53, E131‐M54, L135‐M54, W140‐M54, W140‐I59, W140‐V62, W140‐L94, L142‐I59, L142‐A63, and H143‐A63, with the first position from CCNI, the second from CDK6 (numberings from Uniprot entries ID: Q14094 for CCNI and ID: Q00534 for CDK6). The docking model with lowest energy was submitted to 100 ns of explicit solvent molecular dynamics using the openmm toolkit [22] to relax possible energetic strains built up during the comparative modeling protocol.

Conformational sampling, employing Structure‐Based‐Potential molecular dynamics, was conducted to uncover different binding poses for the CCNI/CDK6 model complex. From the ensemble of structures generated, we analyzed the energy contributions of each residue to the binding energy between the cyclin and CDK partners. The final snapshots of the molecular dynamics runs of each CCNI/CDK complex were explored using structure‐based potential (SBP) molecular dynamics to boost the search of conformational binding modes. The simulations were performed using the sbmOpenMM library for the openmm Toolkit [22]. The SBP trajectories were clustered into 1000 different conformations using root mean square deviations as clustering distances. Afterwards, each of the 1000 centroid structures was energy minimized 10 times with the relax protocol using the Rosetta energy function ref2015 [23], producing ten thousand structures (*N* = 10 000) ensembles for each CCNI/CDK complex. The above‐generated structures were analyzed by first calculating their Boltzmann probabilities, *p*_*i*_, inside the ensemble of *N* conformations:

$$p_{i}=\frac{e^{-E_{i}/\mathrm{kT}}}{Q},$$

where *E*_*i*_ is the ref2015 score of the *i*th structure, *kT* is the characteristic energy partition (set to one rosetta energy unit), and *Q* is the partition function of the respective ensemble:

$$Q=\sum_{i}^{N}e^{-E_{i}/\mathrm{kT}}.$$

Interface binding energies $E_{i}^{b}$ for each complex structure were calculated as the difference in the bound complex score $E_{i}^{\text{complex}}$ and the individual chain scores:

$$E_{i}^{b}=E_{i}^{\text{complex}}-\left(E_{i}^{\text{CCNI}}+E_{i}^{\mathrm{CDK}}\right).$$

The final binding energy predictions, *E*_b_, were estimated as the expectation value of the interface binding energy score using the calculated Boltzmann probabilities:

$$E_{b}=\sum_{i}^{N}p_{i}E_{i}^{b}.$$

Per‐residue contributions were straightforwardly obtained by decomposing the Rosetta energy function into individual residue energy scores.

### 2.12 5‐Bromo‐2′‐deoxyuridine incorporation assay

Cell proliferation was quantitated by measuring 5‐bromo‐2′‐deoxyuridine (BrdU) incorporation during DNA synthesis in cells growing exponentially using the Cell Proliferation ELISA, BrdU, colorimetric kit (Roche, Basel, Switzerland) following the manufacturer's protocol. Cells were incubated with BrdU labeling solution (final concentration of 10 μm) for 2 h. Afterwards, cells were fixed and the DNA denaturated and then incubated with a monoclonal antibody conjugated with peroxidase that binds BrdU in the newly synthesized DNA. Complexes were detected using the 3,3′,5,5′‐tetramethylbenzidine substrate, and the absorbance was measured at 370 nm.

### 2.13 Colony formation assay

Cells were seeded in 6‐well plates 3 days after viral infection. Two weeks later, colony formation assays were performed as described elsewhere [24].

### 2.14 Tumor xenografts

Athymic nude mice female (Hsd:Athymic Nude‐*Foxn1* ^*nu*^) at 5 weeks (Envigo, Gannat, France) of age were orthotopically injected in the mammary fad pat with a total of 3 × 10^6^ MDA‐MB‐231 transduced with empty vector (control group) (*n* = 7) or lentiviral vectors expressing CCND1 (*n* = 7) or CCNI (*n* = 8), soaked in Matrigel (BD Biosciences, San Jose, CA, USA). Tumor growth was monitored for 39 days by measuring tumor width (*W*) and length (*L*) when mice were sacrificed. Tumor volume (mm^3^) was estimated from the formula *V* = π/(6 × *L* × *W*2), and at the mice sacrifice, the tumors were dissected out and weighed (g). Animals were housed in individually ventilated cages on a 12‐h light–dark cycle at 21–23 °C and 40–60% humidity. Mice were allowed free access to an irradiated diet and sterilized water. All mouse experiments were approved by the IDIBELL Animal Care Committee (procedure 9111) and the experiments performed in the IDIBELL Animal Core Facility (no. AAALAC‐1155). Experiments were performed in accordance with the guidelines stated in the International Guiding Principles for Biomedical Research Involving Animals, developed by the Council for International Organizations of Medical Sciences (CIOMS). All animal protocols were reviewed and approved according to regional (Generalitat de Catalunya) Institutional Animal Care and Use Committees.

### 2.15 Gene knockdown

To assess the relevance of *CDK6* expression for CCNI proliferative action, cells were transduced and transfected 72 h later with small interfering RNA (siRNA) against CDK6 (sense: GAUGUUGAUCAACUAGGAAAAAUCT; antisense: AGAUUUUUCCUAGUUGAUCAACAUCUG) or nontargeting siRNA (51‐01‐14‐04; IDT, Coralville, IA, USA) using Lipofectamine 2000 (Thermo Fisher Scientific, Waltham, MA, USA). Cells were detached 48 h later and seeded for colony formation assays.

To assess the knockdown effect on cell cycle, cells were seeded and transfected the following day with negative control or siRNA targeting *CCNI* (SASI_Hs01_00052222; Sigma‐Aldrich), *CCND1* (L‐003210‐00; siRNA SMARTPool; Dharmacon, Lafayette, CO, USA) or *CDK6*.

For cell counting, cells were transduced with control or *CCNI*‐targeting shRNA constructs (Origene; TL314135, Rockville, MA, USA), and cell number was monitored 5 days later. Gene knockdown was assessed by qPCR as described below. Lentiviral vectors were prepared as previously described [14].

### 2.16 RT‐qPCR

Total RNA extraction was carried out using Trizol (Invitrogen, Waltham, MA, USA). cDNA was synthesized from total RNA using a reaction mixture composed of RT buffer (Lucigen, Middleton, WI, USA), dNTP (GeneCraft, Jakarta, Indonesia), Random RT primer (Qiagen), M‐MuLV reverse transcriptase (Lucigen), and RNAse inhibitor (Lucigen). Quantitative PCR was performed using SYBR Green I Master Mix (Bio‐Rad). The obtained values were normalized relative to the housekeeping gene 18S and calculated according to the $2^{-\Delta \Delta C_{t}}$ method. The primers used are listed in Table S2.

### 2.17 Cell synchronization

A549 cells were seeded and transfected the following day with nontargeting siRNA (NT) or with the indicated targeting siRNA. Three days later, cells were synchronized in G0/G1 by serum starvation and released into media containing 10% FBS, and nocodazole. Cells were collected 24 h later for the analysis of cell cycle distribution by propidium iodide as described below.

For western blot analysis, A549 cells were transfected and synchronized in G0/G1 by serum starvation and collected after release into a medium containing 10% FBS at different time points.

### 2.18 Cell cycle analysis by flow cytometry

Cells were plated in 6‐multi well plates and transfected as described above. After 72 h, cells were trypsinized and centrifuged at 200 *g* for 4 min: the culture medium was discarded and the pellet was fixed overnight at −20 °C with a solution of cold 70% ethanol. Cells were then centrifuged at 200 *g* for 4 min and ethanol was discarded. The pellet was resuspended in a solution of PBS containing RNAse (20 μg·mL^−1^) and propidium iodide (50 μg·mL^−1^) and incubated for 45 min in the dark, at 37 °C. The propidium iodide fluorescence was measured on a FACScan flow cytometer (BD FACSCalibur™). The data were gated to exclude cell debris and aggregates and analyzed using the flowing Software 2.5.1 (Turku Centre for Biotechnology, Turku, Finland). The results are expressed as % of total cells.

### 2.19 Depmap and TCGA analyses

Gene expression data from the Depmap portal (depmap.org/, DepMap 20Q2 Public) [25] were used to perform a correlation analysis between the expression of CCNI and all the other genes. The first 1000 genes with a *P*‐value ≤ 1.83 e^−31^ and a Spearman correlation value ≥ 0.3 were selected for a Gene Set Enrichment Analysis (GSEA) using the GSEA portal [26]. For TCGA analyses, data sets were obtained from the Human Protein Atlas website [27].

Samples were ranked considering target gene expression and divided into four even groups. Comparisons were made between samples with the lowest and the highest 25% expression. Box plots display a central rectangle corresponding to the interquartile range and the inside line represents the median. Outliers, defined as data points that were either 1.5 × interquartile range above the third quartile, or 1.5 × interquartile range below the first quartile, were not displayed in the box plots but were considered for *P*‐value calculation [28].

### 2.20 Kaplan–Meier analysis

Patients were divided by the median expression of *CCNI* into low‐ and high‐expression groups and the overall survival was presented as Kaplan–Meier plots [29].

### 2.21 Statistical analysis

Data are presented as the means ± standard errors of the mean (SEMs). Except when indicated otherwise, statistical significance was determined using the Mann–Whitney test with the following categories for *P*‐values: ***P < 0.001; **P < 0.01; and *P < 0.05.

## 3 Results

### 3.1 CDK6 is activated by CCNI

**Fig. 1:**
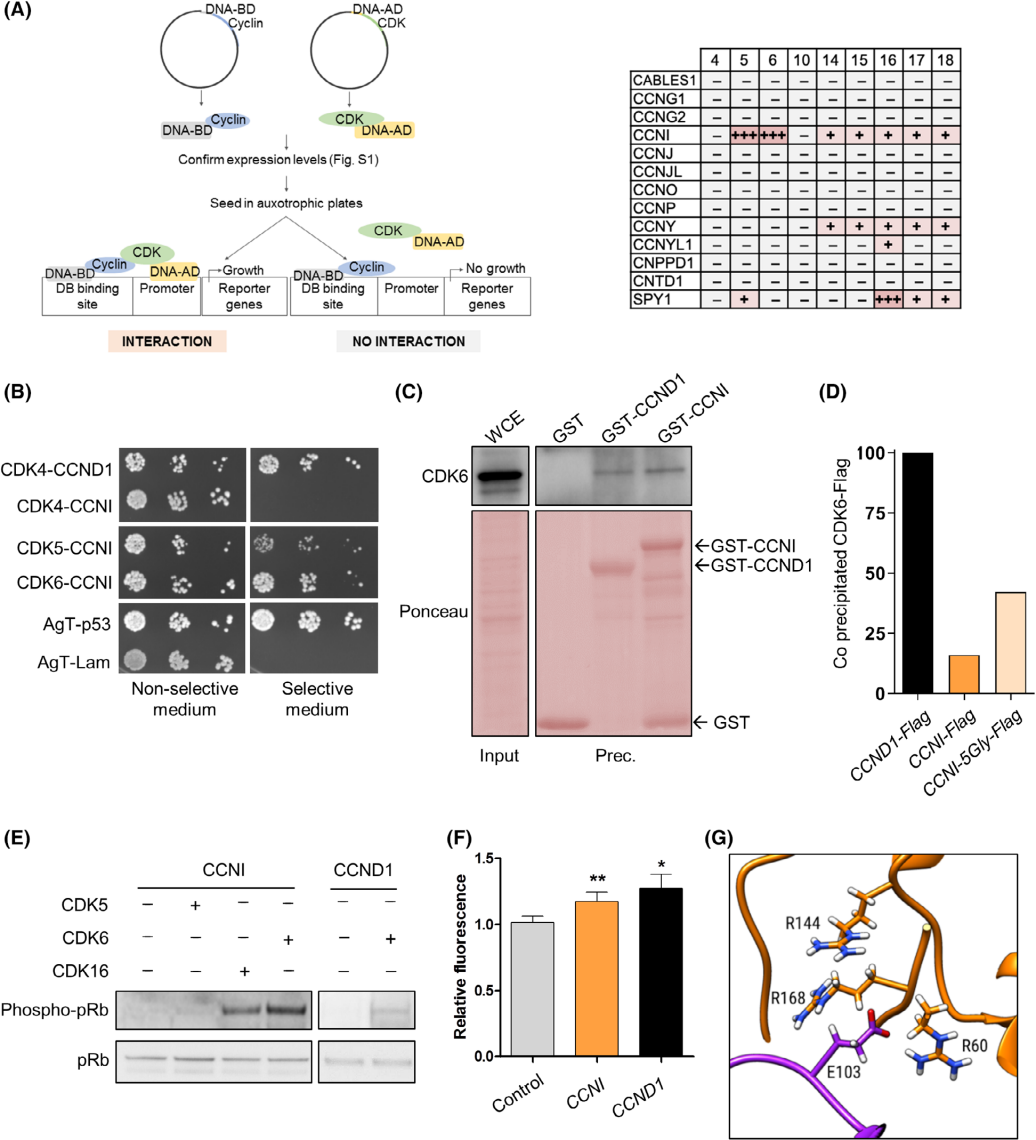
CDK6 is activated by the atypical CCNI. (A) The interactions between atypical cyclins and CDKs were monitored by the yeast two‐hybrid screening. Bait proteins (cyclins) were expressed as Gal4 DNA‐binding domain (DNA‐BD) fusion proteins, whereas prey proteins (CDKs) were expressed fused to the Gal4 activation domain (AD). When bait and prey interact, the DNA‐BD and AD are brought into proximity and activate the transcription of auxotrophic markers, such as HIS and ADE, enabling yeast growth in media lacking those amino acids. The table shows the positive CDK–Cyclin interactions detected, which are highlighted in pink, and represents the results from three independent experiments. (B) Representative images of two independent experiments of the yeast two‐hybrid assay showing CCNI interactions with CDK4, CDK5, and CDK6. AgT‐p53 was used as positive control, whereas AgT‐Lam was used as negative control. (C) Trapping assay. EBV‐immortalized lymphocytes were incubated with GST or with GST‐CCNI purified from *Escherichia coli*. CDK6 was detected by western blot using a specific antibody, whereas recombinant proteins were detected using an anti‐GST. The image is representative of two independent experiments. (D) Immunoprecipitation assay. A549 cells were transduced with empty lentiviral vector or with the indicated Flag‐tagged cyclins, which were immunoprecipitated using an anti‐Flag antibody. The graph shows the quantitation of a representative experiment of two independent experiments after normalization with the amount of precipitated protein. The amount of CDK6 that precipitates with CCND1‐Flag, which was used as a positive control, was considered 100%. (E) *In vitro* kinase assay using recombinant CDKs and CCNI and pRb as substrate. The quantitation of at least two independent experiments is shown in Fig. S5 (*n* = 3 for CCNI‐CDK5 and CCNI‐CDK6; *n* = 2 for the other combinations). (F) Kinase assays using the CDK6 phosphorylation biosensor (TAMRA‐labeled CDKACT6) and protein extracts obtained from MDA‐MB‐231 cells transduced with the indicated constructs. The relative fluorescence of the assay at 6000 s is shown. The columns represent the means ± SEMs of seven independent experiments performed in triplicates. *P < 0.05, **P < 0.01 vs control, paired *t*‐test. (G) Predictive model of CCNI residue E103 interaction with three CDK6 arginines (R60, R144, and R168). CCNI is shown in purple and CDK6 in orange.

First, we monitored the ability of atypical cyclins to interact with CDKs belonging to different subfamilies using the yeast two‐hybrid assay [30] (Fig. 1A). We also included in this screen SPY1, which is not considered a cyclin but is able to interact with CDKs through the ‘RINGO’ box domain [4, 31]. After confirming the adequate expression of all proteins (Fig. S1A,B), we have only detected 17 interactions out of 117 analyzed couples (Fig. 1A), suggesting that the screen was highly stringent. Among these interactions, 7 had already been validated: CDK5‐SPY1 [32], CDK5‐CCNI [33, 34], CDK14‐CCNY [12], and CDK16‐CCNI/CCNY/CCNYL1/SPY1 [24, 35]. We have also identified 10 new complexes involving SPY1 (with CDK17 and CDK18), CCNI (with CDK6, and with less affinity with CDK14, CDK15, CDK17, and CDK18), and CCNY (with CDK15, CDK17, and CDK18). Among the interactions uncovered by our screen, we found particularly remarkable the fact that CCNI interacts with CDK6 but does not seem to interact with CDK4 (Fig. 1B). In line with these findings, the very recent results from the Human Reference Protein Interactome Mapping Project (http://www.interactome‐atlas.org/) [36] suggest that CCNI interacts with CDK5, CDK6, and CDK3 but not with CDK4. Still, further experimental evidence is required to rule out an interaction between CDK4 and CCNI.

We validated the two more significant interactions identified in the previous screen: the SPY1–CDK16 and the CCNI–CDK6 complexes. To confirm the interaction between SPY1 and CDK16, we performed an immunoprecipitation in HEK293T cells overexpressing *SPY1* with an anti‐Flag antibody. Western blot analysis using an antibody against CDK16 demonstrated that CDK16 coimmunoprecipitates with SPY1 (Fig. S2A). Next, we conducted an *in vitro* substrate‐trapping assay in extracts obtained from A549 and U87 cells using GST‐fused SPY1. SPY1 precipitated with CDK16 in both cell line extracts (Fig. S2B,C), confirming the previous results.

One of the most significant interactions unveiled by the screening was that detected between CCNI and CDK6. To confirm the interaction, we performed a trapping assay in EBV‐immortalized lymphocytes, which display a high level of both *CDK6* and *CCNI* expression (Fig. S3). In line with the results of the yeast 2‐hybrid assay, CDK6 precipitated with recombinant GST‐CCNI (Fig. 1C), in comparable amounts to GST‐cyclin D (CCND1), a well‐known CDK6 partner. We chose CCND1 as a positive control, considering the substantial evidence supporting its role in cancer and the greater depth of CCND1 functional characterization compared with CCND2 and CCND3. Similar results were obtained in rat whole brain extract, in which CCNI plays a physiological role [37] (Fig. S4A). Afterwards, we transduced A549 cells with an empty lentiviral vector or with a CCNI‐overexpressing construct and the flag‐tagged protein was immunoprecipitated with an anti‐Flag antibody. Despite the lower level of precipitated CCNI as compared to CCND1, CDK6 precipitated with CCNI‐Flag (Fig. 1D; Fig. S4B). This result was recapitulated using a *CCNI* construct with a different tag, although this interaction appears to be weaker than the CDK6‐CCND1 interaction, at least in our experimental conditions (Fig. 1D; Fig. S4B).

Considering that the CDK6–CCND1 complex phosphorylates the tumor suppressor pRb to drive G_1_‐ to S‐phase transition, we then sought to demonstrate that the CDK6–CCNI complex was active. For this purpose, we conducted an *in vitro* kinase assay and compared the potency of CCNI when bound to the three partner CDKs detected in our screen to phosphorylate pRb. The CDK6–CCNI complex was able to phosphorylate pRb, similarly to the CCND1–CDK6 complex (Fig. 1E; Fig. S5). In line with previous reports that show that CDK16 phosphorylates pRb at Ser780 [38], we have also observed pRb phosphorylation in the presence of the CDK16–CCNI complex (Fig. 1E; Fig. S5). To investigate the activity of the CDK6–CCNI complex in human cells, we used a peptide biosensor that reports on CDK6 activity through changes in fluorescence [15]. Importantly, the signal specifically reflects CDK6 activity as it has been demonstrated that CDK4 (the most closely related to CDK6 with a 70% homology) cannot bind and phosphorylate the substrate of CDKACT6; this way, the biosensor specifically reports CDK6 activity in complex and physiologically relevant environments [15]. Extracts from MDA‐MB‐231 cells overexpressing *CCNI* present a higher fluorescent signal than control, similar to *CCND1*, consistent with an increase in CDK6 activity (Fig. 1F).

Whereas the cyclin boxes of CCNI and CCND1 share a high degree of homology (Fig. S6A), the rest of the sequence is less similar, complicating the predictions regarding the structure of the new complex. Therefore, we explored the conservation of common interface positions of other cyclin/CDK complexes. From 577 PDB structures containing cyclin structures, we found only 10 containing unique cyclin/CDK complexes, with 11 and 9 conserved positions at the cyclin and kinase partners, respectively (Fig. S6B), including Lys100 and Glu131, which are conserved in all cyclins [4]. Remarkably, among the 16 residues that contribute with more than 0.5% of the total binding energy of the CCNI–CDK6 complex, we found 9 of the 11 CCNI residues of the common interface positions (Fig. S6C), confirming that CCNI interacts with CDK6 via a canonical cyclin box‐CDK‐binding mode [39]. Nevertheless, our analysis suggests that there are other important residues, such as E103, which pertains to a special insertion of four negatively charged positions, and E^103^EDE^106^, a motif shared by the atypical cyclins CCNI2 and CNPPD1. CCNI E103 might be interacting with a group of three positively charged residues in CDK6, R60, R144, and R168 (Fig. 1G), which are found in several CDK6 PDB structures adopting a conformation similar to the one of our model (Fig. S6D). Moreover, we show by yeast two‐hybrid assay that either CCNI E103 or CDK6 R60 mutation disrupts CCNI–CDK6 interaction, supporting that these residues are indeed critical for the formation of this complex (Fig. S6E).

### 3.2 Overexpression of *CCNI* significantly promotes breast cancer growth *in vitro* and *in vivo*

**Fig. 2:**
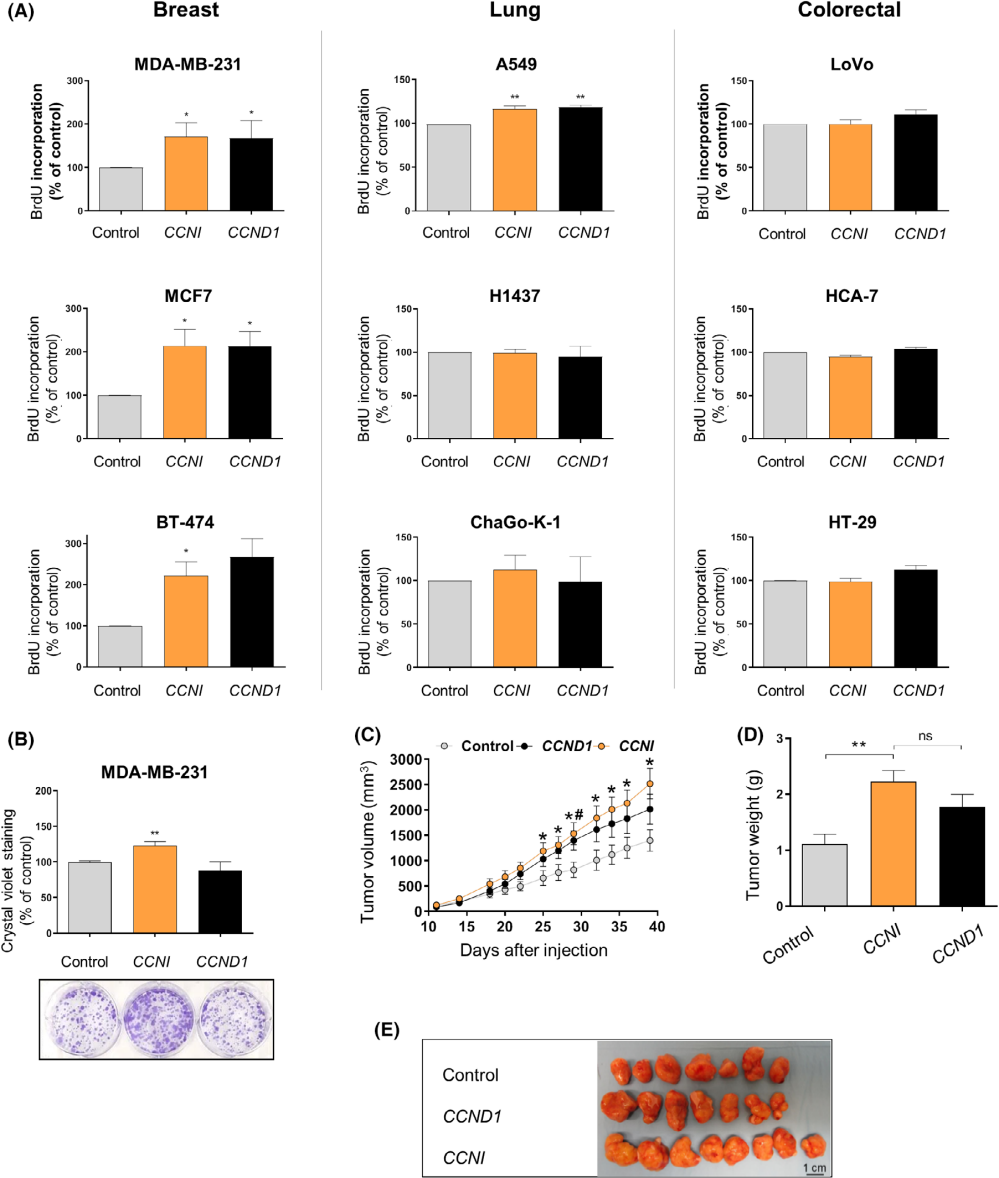
Overexpression of *CCNI* significantly promotes breast cancer growth *in vitro* and *in vivo*. (A, B) Cells were transduced with empty lentiviral vector (control) or with the indicated cyclin‐overexpressing construct. (A) Cell proliferation was evaluated by BrdU incorporation assays 5 days later. Columns represent the mean ± SEM of at least three independent experiments performed in triplicates (*n* = 4 for MDA‐MB‐213, MCF7, BT‐474, and A549; *n* = 3 for H1437, ChaGo‐K‐1, LoVo, HCA7, and HT‐29). *P < 0.05, **P < 0.01 vs control, Mann–Whitney test. (B) Efficiency of cell colony formation. Columns represent the mean ± SEM of four independent experiments performed in duplicates. Image below the graph shows representative images of colony formation assays. **P < 0.01 vs control, Mann–Whitney test. (C–E) Mice were orthotopically injected into mammary fat pads with MDA‐MB‐231 with empty vector (control) (*n* = 7), *CCND1* (*n* = 7), or *CCNI* (*n* = 8). (C) The tumor volumes were measured at the indicated number of days. *P < 0.05, CCNI vs control; ^#^ *P* < 0.05, CCND1 vs control, Mann–Whitney test. (D) Tumor weight at day 39. **P < 0.01, vs control; ns, not significant, Mann–Whitney test. (E) Representative images of the tumors resected at day 39. The scale bar shown corresponds to 1 cm.

To investigate the effect of endogenous CCNI on cancer cell proliferation, cell lines representative of the three most frequent cancer types (breast, lung, and colon) were transduced with lentiviral vectors expressing *CCNI* or *CCND1* (Fig. S7) and BrdU incorporation was monitored. We preferred to use overexpression to better mimic a subset of human cancers that present *CCNI* upregulation. *CCNI* overexpression significantly increased the proliferation of MDA‐MB‐231, MCF7, and BT‐474 breast cancer cells, as well as A549 non‐small lung cancer cells (Fig. 2A), while no effect was observed on any of the other five cell lines. Likewise, *CCNI* overexpression increased the clonogenicity of MDA‐MB‐231 and A549 cells (Fig. 2B; Fig. S8). Noteworthy, the panel of 9 cell lines shows that CCND1 increases cell proliferation only in a subset of cell lines; we find remarkable the fact that CCNI only increases proliferation, and with the same efficiency (Mann–Whitney test), in the exact same cell lines (Fig. S9). These similarities are unlikely to be a coincidence and suggest that CCNI and CCND1 act in the same specific genetic backgrounds through a common mechanism.

To investigate the effect of *CCNI* expression *in vivo*, we selected MDA‐MD‐231 cells, a model of triple‐negative breast cancer, which has a poorer prognosis as compared to other breast cancer types. MDA‐MD‐231 cells overexpressing *CCNI* formed significantly faster‐growing tumors than control (Fig. 2C–E), supporting that *CCNI*, when overexpressed, contributes to enhance tumor growth.

### 3.3 CCNI increases cancer cell proliferation through its interaction with CDK6

**Fig. 3:**
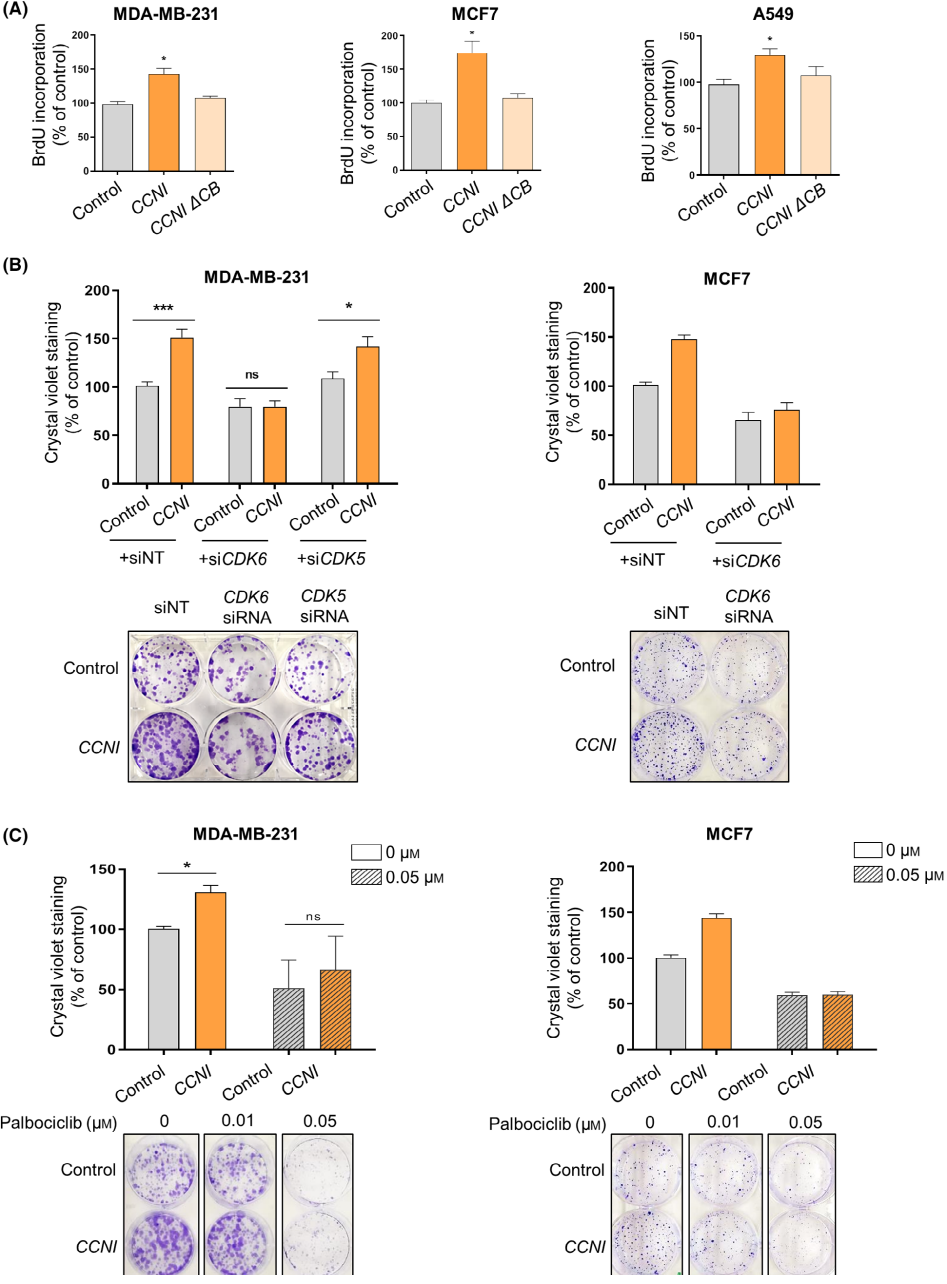
CCNI promotes cancer cell proliferation through the activation of CDK6. (A) Cells were transduced with empty vector (control) or with the lentiviral vector expressing wild‐type or CDK‐binding‐deficient CCNI (CCNI ΔCB). Columns represent the mean ± SEM of four experiments performed in duplicates. *P < 0.05 vs control, Mann–Whitney test. (B) Cells were transduced with empty vector (control) or with the lentiviral vector expressing *CCNI*. Cells were then transfected with nontargeting siRNA (siNT) or with the indicated targeting siRNA and, 48 h later, cells were seeded to assess colony formation 2 weeks later. Columns represent the mean ± SEM of at least four independent experiments performed in duplicates. *P < 0.05, ***P < 0.001 vs the indicated control, Mann–Whitney test. ns, not significant. (C) Cells were transduced with empty vector (control) or with the lentiviral vector expressing *CCNI*. Cells were then seeded in 6‐well plates and treated with the indicated concentrations of palbociclib. Colony formation was monitored 2 weeks later. Columns represent the mean ± SEM of four experiments performed in duplicates. *P < 0.05 vs the indicated control, Mann–Whitney test. ns, not significant.

Next, we investigated whether CCNI proliferative actions are CDK‐dependent. For this purpose, we selected three cell lines in which *CCNI* upregulation promotes a robust increase in cell proliferation (A549, MCF7, MDA‐MB‐231) and transduced them with lentiviral vectors expressing wild‐type *CCNI* and a mutant form of *CCNI* lacking the cyclin box motif, which is required for CDK binding. The CDK‐binding‐deficient form of CCNI was unable to increase cell proliferation, in contrast to the wild‐type form, suggesting that CCNI stimulates cancer cell proliferation through the interaction with a CDK (Fig. 3A). Considering that, according to the yeast two‐hybrid screen (Fig. 1A), CCNI interacts with several CDKs, we next examined whether CCNI‐induced clonogenicity was dependent on *CDK6* expression and activity. *CDK6* silencing prevented CCNI‐induced clonogenicity in MDA‐MB‐231, MCF7 (Fig. 3B; Fig. S10A), BT‐474, and A549 cells (Fig. S10B). Noteworthy, CCNI‐induced clonogenicity in MDA‐MB‐231 cells was not abrogated by *CDK5* silencing, suggesting that the interaction with CDK6 is the one critical to promote cell proliferation (Fig. 3B). Interestingly, *CCNI* overexpression seems to lead to a decline in *CDK5* expression (Fig. S10A); although we have not explored this result, it may indicate that CCNI regulates CDK5 through a feedback mechanism. Treatment with the CDK4/6 inhibitor palbociclib also abrogated the increase in cell clonogenicity promoted by CCNI in MDA‐MB‐231, MCF7 (Fig. 3C), BT‐474, and A549 cells (Fig. S10C), which is compatible with an effect mediated via CDK activation.

### 3.4 *CCNI* overexpression activates the expression of E2F‐regulated genes

**Fig. 4:**
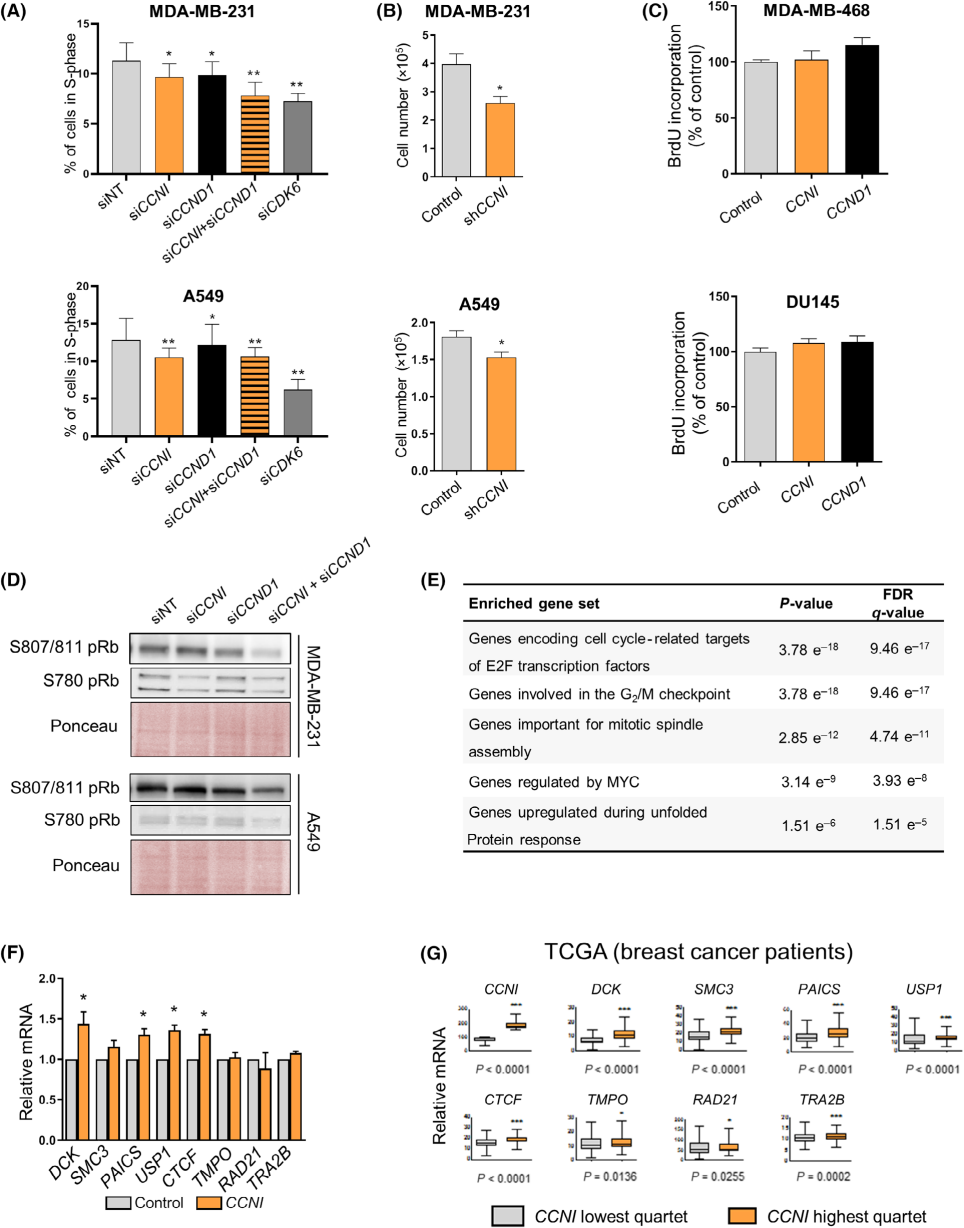
*CCNI* overexpression promotes E2F‐regulated gene expression. (A) The percentage of cells in S phase was analyzed by FACS in A549 and MDA‐MB‐231 cells transfected with nontargeting control (siNT), *CDK6*, *CCNI*‐ or *CCND1*‐targeting siRNA, and a combination of both. Columns represent the mean ± SEM of six (MDA‐MB‐231) or five (A549) independent experiments. *P < 0.05, **P < 0.01 vs control, Mann–Whitney test. (B) Cells were transduced with empty (control) or *CCNI*‐targeting shRNA and cell number was assessed 5 days later. Columns represent the mean ± SEM of four experiments performed in duplicates. *P < 0.05 vs control, Mann–Whitney test. (C) Retinoblastoma protein (pRb) null cancer cells were transduced with empty vector (control) or with the lentiviral vector expressing *CCNI* or *CCND1* and BrdU incorporation was monitored 5 days later. Columns represent the mean ± SEM of three independent experiments performed in triplicate. No significant differences were found as compared to control, Mann–Whitney test. (D) Phosphorylation of pRb in A549 and MDA‐MB‐231 cells was monitored after transfection with nontargeting control (siNT), *CCNI*‐ or *CCND1*‐targeting siRNA, and a combination of both. A representative image of three independent experiments is shown. (E) A correlation analysis between *CCNI* expression and each of the genes in the Depmap portal (depmap.org/) was carried out. The 1000 highest ranked genes (Spearman correlation coefficient < 1 × 10^−22^) were submitted to a GSEA. To validate the enrichment analysis of F, GSEA portal uses the cumulative hypergeometric distribution as described in [51]. (F) MDA‐MB‐231 cells were transduced with empty (control) or *CCNI*‐expressing construct and gene expression was monitored by RT‐qPCR. The obtained values were normalized relative to the housekeeping gene *RNA18SN5*. Columns represent the mean ± SEM of four experiments performed in duplicates. *P < 0.05 vs control, Mann–Whitney test. (G) Data from TCGA were used to assess the correlation between *CCNI* and E2F‐regulated gene expression profiles in breast cancer patients (*n* = 269 for each quarter). Error bars indicate minimum/maximum values. *P < 0.05, ***P < 0.001 vs *CCNI* lowest quarter, Mann–Whitney test. Data obtained from the Human Protein Atlas database on June 18, 2020.

The results obtained so far place CCNI, in partnership with CDK6, at the G_1_‐ to S‐phase transition regulation; therefore, it is reasonable to expect that *CCNI* downregulation will affect the cell cycle. Indeed, *CCNI* knockdown significantly decreased the percentage of MDA‐MB‐231 and A549 cells reaching the S phase, similarly to cyclin D (Fig. 4A; Fig. S11A). Interestingly, the effect of *CCNI* and *CCND1* downregulation on MDA‐MB‐231 cells was additive, and similar to the effect of *CDK6* knockdown (Fig. 4A). On the other hand, in the case of A549, the knockdown of *CDK6* led to a more pronounced effect than the knockdown of both cyclins, suggesting that other D‐type cyclins may be able to activate CDK6 in the absence of CCNI or CCND1 (Fig. 4A). Cell number was also significantly reduced after *CCNI* downregulation (Fig. 4B; Fig. S11B). Considering that CDK6 acts through pRb phosphorylation, CCNI proliferative actions should require pRb expression. Interestingly, neither CCNI nor CCND1 significantly increased the proliferation of ChaGo‐K‐1 cells, which lack *RB1* expression (Fig. 2A; Fig. S9). This observation was further confirmed in two additional pRb‐null cellular models (Fig. 4C; Fig. S12). We then investigated the effect of *CCNI* downregulation on two pRb phosphorylation sites in cancer cell lines in which the pRb checkpoint is active. *CCNI* downregulation decreased S780 pRb phosphorylation in MDA‐MB‐231 cells, whereas in A549 only the knockdown of both *CCNI* and *CCND1* significantly impaired pRb phosphorylation (Fig. 4D). Likewise, a discernible effect on S807/811 pRb phosphorylation in either A549 or MDA‐MB‐231 cells was only observed after silencing both *CCNI* and *CCND1* (Fig. 4D), supporting the idea of some additivity in the regulation of pRb phosphorylation. Furthermore, we extracted data from the Depmap portal (depmap.org/, DepMap 20Q2 Public) [25], which contains gene expression profiles of 1304 cell lines of different histological types, and investigated which genes are coexpressed with endogenous *CCNI*. First, we generated a list of genes ordered by how closely their expression correlated with that of *CCNI* across all the cell lines. The first 1000 genes were selected for a GSEA using the GSEA portal and the hallmark gene set collection of the Molecular Signatures Database (MSigDB) [26]. The top hit is a list of 34 genes regulated by E2F transcription factors (*P*‐value = 10^−18^, FDR = 10^−17^) (Fig. 4E), supporting a model where the CCNI–CDK6 complex phosphorylates pRb to unleash E2F‐dependent transcription.

To confirm that the expression correlations obtained from the DepMap portal are a consequence of CCNI upregulation, we randomly selected eight genes from the list of 34 genes regulated by E2F transcription factors, all of them considered cell cycle genes according to the Target Gene Regulation Database [40], and analyzed their expression by RT‐qPCR in MDA‐MB‐231 cells overexpressing CCNI (Fig. 4F); we have also included in this analysis 15 other genes that are well‐established E2F targets (Fig. S13). We found that *CCNI* upregulation led to significantly higher expression of several E2F target genes (Fig. 4F; Fig. S13). To investigate whether the results obtained in cell lines can be extrapolated to human cancer, we exploited the RNA‐seq database from The Cancer Genome Atlas (TCGA). We grouped breast cancer patients with the lowest and highest quarters of *CCNI* expression and we found that *CCNI* was significantly linked to the expression of several E2F‐regulated genes in breast cancer patients (Fig. 4G).

Altogether, these results suggest that CCNI activates CDK6 to promote pRb phosphorylation and E2F‐mediated gene expression, similar to CCND1. Therefore, considering that CCND1 is implicated in cell cycle entry after serum starvation, we hypothesized that CCNI might be playing a role in these circumstances as well. Indeed, in synchronized A549 cells, *CCNI* downregulation led to a delay in cell cycle entry (Fig. S14A). In line with these findings, the analysis of pRb phosphorylation after serum starvation showed that *CCNI* downregulation decreased pRb phosphorylation, an effect that was more prominent when both cyclins were silenced (Fig. S14B).

Finally, we investigated *CCNI* expression in human cancer in UCSC Xena [41]. Several primary tumors of different histological types display higher *CCNI* mRNA levels as compared to normal tissues (Fig. S15). These observations are in line with the demonstration that *CCNI* is overexpressed at the protein level in lung cancer patients [14] and that salivary *CCNI* mRNA levels can be used to discriminate between lung cancer patients and healthy individuals [41]. Moreover, the analysis of the overall survival of cancer patients stratified by *CCNI* expression level suggests that this cyclin may have prognostic significance in cancer. Depending on the histological type, *CCNI* may be associated with either a better (sarcoma, breast) or worse (renal clear cell carcinoma, ovarian) clinical prognosis (Fig. S16A). Within the same histological type, the effects of CCNI may also depend on the genetic background, as highlighted by the opposing impact of *CCNI* on gastric and ovarian patient survival with ERBB2 or p53 mutations, respectively (Fig. S16B). The prognostic significance of *CCNI* within the same tumor type may also depend on smoking or alcohol consumption (Fig. S16C). Therefore, and similarly to other cyclins, the molecular effects of CCNI seem to be intrinsically linked to tissue, genetic and environmental contexts.

## 4 Discussion

Our yeast two‐hybrid screen suggests that the subfamily of atypical cyclins can be divided into two different groups: those that act through CDK activation and those that act independently of CDK binding (Fig. 1A). An important limitation of the yeast two‐hybrid assay is that toxicity may limit protein expression; other factors, such as the absence of a specific post‐translational modification or the lack of a third partner that enables complex assembly may also hamper the detection of complexes. Therefore, while it is important not to overinterpret negative results, our results suggest that most atypical cyclins do not interact with a CDK and would be included in the latter group (Fig. 1A). Although some canonical cyclins are reported to perform a few actions without CDKs [42], the fact that atypical cyclins mainly act through alternative mechanisms to CDK activation is remarkable and confirms this particularity as a defining trait of the subfamily of atypical cyclins. Notable exceptions to this rule are CCNYL1, CCNY, and CCNI, with the latter two interacting with several atypical CDKs.

Until now, CCNI was mostly known for its interaction with CDK5 [9], which is mainly expressed in brain (Fig. S3). Here, we show that CCNI also acts as a CDK6 activator. Interestingly, while CCNI is relatively ubiquitous, high levels of CDK6 expression are only found in highly proliferative tissues, such as EBV‐immortalized lymphocytes or cultured fibroblasts (Fig. S3). It is then possible that CCNI plays specific roles in different tissues through the activation of different CDKs.

According to the standing model of cell cycle regulation, CDK6 associates exclusively with D‐type cyclins to phosphorylate pRb, releasing E2F transcription factors and driving G_1_‐ to S‐phase transition [43, 44]. Although the CCNI–CDK6 interaction seems to be weak and its detection by immunoprecipitation was technically challenging (Fig. 1D; Fig. S4), we provide several evidences that support this claim. We demonstrate that the CCNI–CDK6 complex phosphorylates pRb (Fig. 1E). CCNI, like CCND and viral cyclins, has the defined LxCxE motif required for interaction with pRb LxCxE cleft [45, 46]. Remarkably, the group of Skotheim has demonstrated that pRb has an additional docking site in its C‐terminal alpha‐helix that interacts with an unspecified region of CCND [1], enabling a specific pattern of pRb phosphorylation that is different from the one promoted by other cyclins. Therefore, it remains to be established whether CCNI may also interact with the C‐terminal alpha‐helix of pRb as CCND does.

In line with pRb phosphorylation, we found a positive correlation between CCNI and E2F target gene expression (Fig. 4E–G; Fig. S13) and an increase in cell proliferation (Fig. 2), in agreement with previous reports that implicated CCNI in the proliferation of HeLa [47] and A549 cells [14]. While some of these phenotypes could be mediated by binding to other CDKs, the results of the two‐hybrid screen and the *in vitro* phosphorylation assays point CDK6 as the most relevant interactor of CCNI (Fig. 1A,E). Moreover, CCND1 and CCNI promoted cell proliferation with comparable efficiency but only if pRb was present in the genetic background of the cell line (Figs 2 and 4C). In cell lines where overexpression of *CCND1* did not elicit any change in proliferation, the same was true for *CCNI* overexpression. This remarkable coincidence suggests that both proteins share a common underlying molecular mechanism, the regulation of pRb phosphorylation. Such regulation seems to be additive, as silencing each cyclin separately in asynchronous cultures led to a decrease in pRb phosphorylation levels, but the double knockdown drastically reduced the amount of phosphorylated pRb (Fig. 4D). The same genetic approach demonstrated that the combined silencing of both cyclins produced an additive effect on the number of cells in S phase (Fig. 4A). One of CDK6 critical functions is to regulate cell cycle resume from quiescent states induced by, for instance, serum deprivation. We have shown that *CCNI* silencing in quiescent cells led to a delay in cycle reentry following serum addition (Fig. S14A) and a decrease in pRb phosphorylation levels (Fig. S14B). This decline was again additive when both cyclins were simultaneously silenced (Fig. S14B).

Therefore, we propose that CCNI and CCND1 operate in tandem during the G1 phase to facilitate pRb phosphorylation and E2F‐mediated gene expression and that such additivity may enhance the robustness of cell cycle control under normal conditions (i.e., physiological tissue regeneration). However, it may also increase the potential for cancer development by providing multiple opportunities for mutations that enable cells to bypass normal cell cycle checkpoints and promote uncontrolled cell proliferation.

Finally, we cannot rule out that both cyclins play more specific roles under certain conditions or in certain tissues. Whereas it is known that CCND1 responds to nutrient availability through the Ras/Erk pathway, the regulation of CCNI is not well understood, and further research is necessary to determine whether both cyclins are redundantly controlled by growth factors or whether they respond to different stimuli. Given the multiple contexts that may be found in the highly heterogeneous tumor environment, a better understanding of CCNI regulation will be critical to unveil the full significance of the CCNI and CCND1 interplay.

Interestingly, we show that the effects of CCNI on the growth of animal models of triple‐negative breast cancer are similar to the ones promoted by CCND1, the deregulation of which is a breast cancer hallmark [48] (Fig. 2C–E). Given the poor prognosis and lack of effective treatments for triple‐negative breast cancer patients, CCNI may represent an innovative prognostic marker and drug target in this malignancy. Moreover, the oncogenic role of CCNI may eventually extend to other malignancies. Indeed, our group has demonstrated that *CCNI* is overexpressed in lung cancer patients and it is associated with a worse clinical prognosis [14], while others have reported that salivary *CCNI* mRNA could be used to discriminate lung cancer patients from normal subjects [49]. Furthermore, these observations support that CCNI is implicated in cancer progression and has the potential to become a biomarker for patient stratification (Fig. S16). Interestingly, CCND1 levels are significantly correlated with patient survival in a limited number of tumor types (Fig. S16D). Considering that cyclins are regulated at post‐translational level, it is conceivable that the prognostic significance based on mRNA levels underestimates their clinical impact.

Finally, the existence of another cyclin that is able to activate CDK6 opens new avenues regarding the use of CDK4/6 inhibitors in clinical practice. Until now, the only marker for CDK4/6 inhibitors employed in decision‐making is the loss of pRb function, which is relatively uncommon; by contrast, genetic alterations of *CCND1* are more common, but their predictive value has been difficult to establish [50]. The identification of the CCNI–CDK6 complex may enable a better understanding of the tumor genetic landscape that determines sensitivity to CDK4/6 inhibition.

## 5 Conclusions

We show that CCNI activates CDK6 to increase cancer cell proliferation via pRb phosphorylation, suggesting that CCNI is another piece in the cell cycle machinery puzzle and a new potential oncogenic driver.

## Conflict of interest

The authors declare no conflict of interest.

## Author contributions

EQ, NM, SH‐O, AVillanueva, MPCR, and JC contributed to conceptualization; EQ, NM, SH‐O, AS‐B, LG, AF‐E, SP, JMM‐L, SB, MCM, PMM‐T, MF, JV‐F, AVidal, and AVillanueva contributed to investigation; MPCR and JC contributed to writing and supervision; JC and JV‐F contributed to funding acquisition; MCM contributed to resources.

### Peer review

The peer review history for this article is available at https://www.webofscience.com/api/gateway/wos/peer‐review/10.1002/1878‐0261.13438.

## Supporting information


**Fig. S1.** Cyclin and CDK protein expression in yeast was confirmed by western blot.
**Fig. S2.** Validation of the CDK6–SPY1 interaction.
**Fig. S3.** CCNI, CDK6, and CDK5 expression in different tissues according to the GTEXPortal (https://www.gtexportal.org/home/).
**Fig. S4.** Trapping and immunoprecipitation assays.
**Fig. S5.** Quantitation of the *in vitro* kinase assay shown in Fig. 1E using pRb as substrate.
**Fig. S6.** Structural analysis of CCNI.
**Fig. S7.** Overexpression of CCNI and CCND1 in cancer cells.
**Fig. S8.** Colony formation assays in A549 and Lovo cells.
**Fig. S9.** Expression of cell cycle regulators in the cell lines displayed in Fig. 2A.
**Fig. S10.** CDK6 silencing or inhibition abrogates CCNI‐induced cancer cell proliferation.
**Fig. S11.** Control of cyclins downregulation.
**Fig. S12.** CCNI overexpression in pRb‐null cell lines.
**Fig. S13.** Effect of CCNI upregulation on the expression of E2F target genes.
**Fig. S14.** Effect of CCNI downregulation on cell cycle entry.
**Fig. S15.** CCNI expression in normal and primary tumor tissues.
**Fig. S16.** The prognostic significance of CCNI depends on the disease context.
**Table S1.** List of antibodies used.
**Table S2.** List of primers used.

## Data Availability

The data that support the findings of this study are available from the corresponding authors (jclotet@uic.es; mpontecardosoribeiro@uic.es) upon reasonable request.
