# Supplementary material for: CDK6 is activated by the atypical cyclin I to promote E2F‐mediated gene expression and cancer cell proliferation
Source: Mol Oncol. 2023 May 31;17(7):1228–45. doi: 10.1002/1878-0261.13438 (PMC10323892; doi:10.1002/1878-0261.13438)
Supplement: Supplementary file 1 — Fig. S1. Cyclin and CDK protein expression in yeast was confirmed by western blot. Fig. S2. Validation of the CDK6–SPY1 interaction. Fig. S3. CCNI, CDK6, and CDK5 expression in different tissues according to the GTEXPortal (https://www.gtexportal.org/home/). Fig. S4. Trapping and immunoprecipitation assays. Fig. S5. Quantitation of the in vitro kinase assay shown in Fig. 1E using pRb as substrate. Fig. S6. Structural analysis of CCNI. Fig. S7. Overexpression of CCNI and CCND1 in cancer cells. Fig. S8. Colony formation assays in A549 and Lovo cells. Fig. S9. Expression of cell cycle regulators in the cell lines displayed in Fig. 2A. Fig. S10. CDK6 silencing or inhibition abrogates CCNI‐induced cancer cell proliferation. Fig. S11. Control of cyclins downregulation. Fig. S12. CCNI overexpression in pRb‐null cell lines. Fig. S13. Effect of CCNI upregulation on the expression of E2F target genes. Fig. S14. Effect of CCNI downregulation on cell cycle entry. Fig. S15. CCNI expression in normal and primary tumor tissues. Fig. S16. The prognostic significance of CCNI depends on the disease context. Table S1. List of antibodies used. Table S2. List of primers used. [file MOL2-17-1228-s001.pdf]

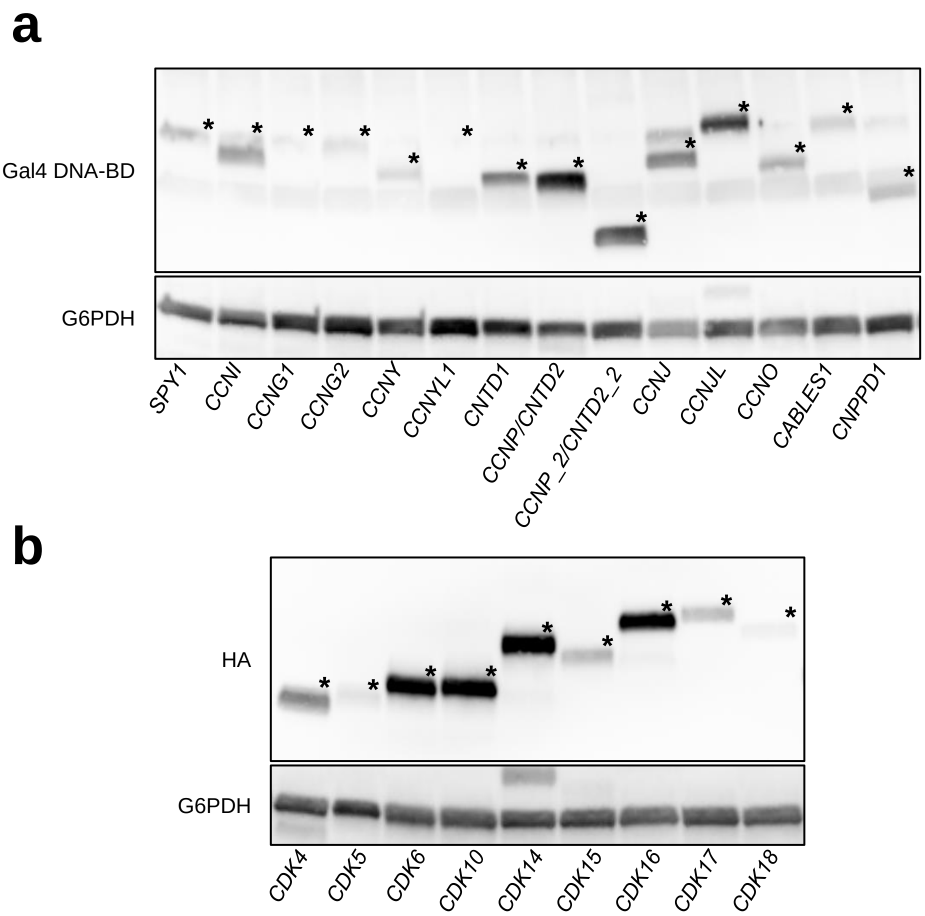

Supplementary Figure 1. Cyclin and CDK protein expression in yeast was confirmed by western blot. (A) Cyclin expression in the yeast two-hybrid screening. was monitored using an anti-Gal4 DNA-BD antibody. (B) CDK expression was confirmed using an anti-HA antibody. G6PDH was used as loading control. Asterisks indicate the bands corresponding to the protein of interest.

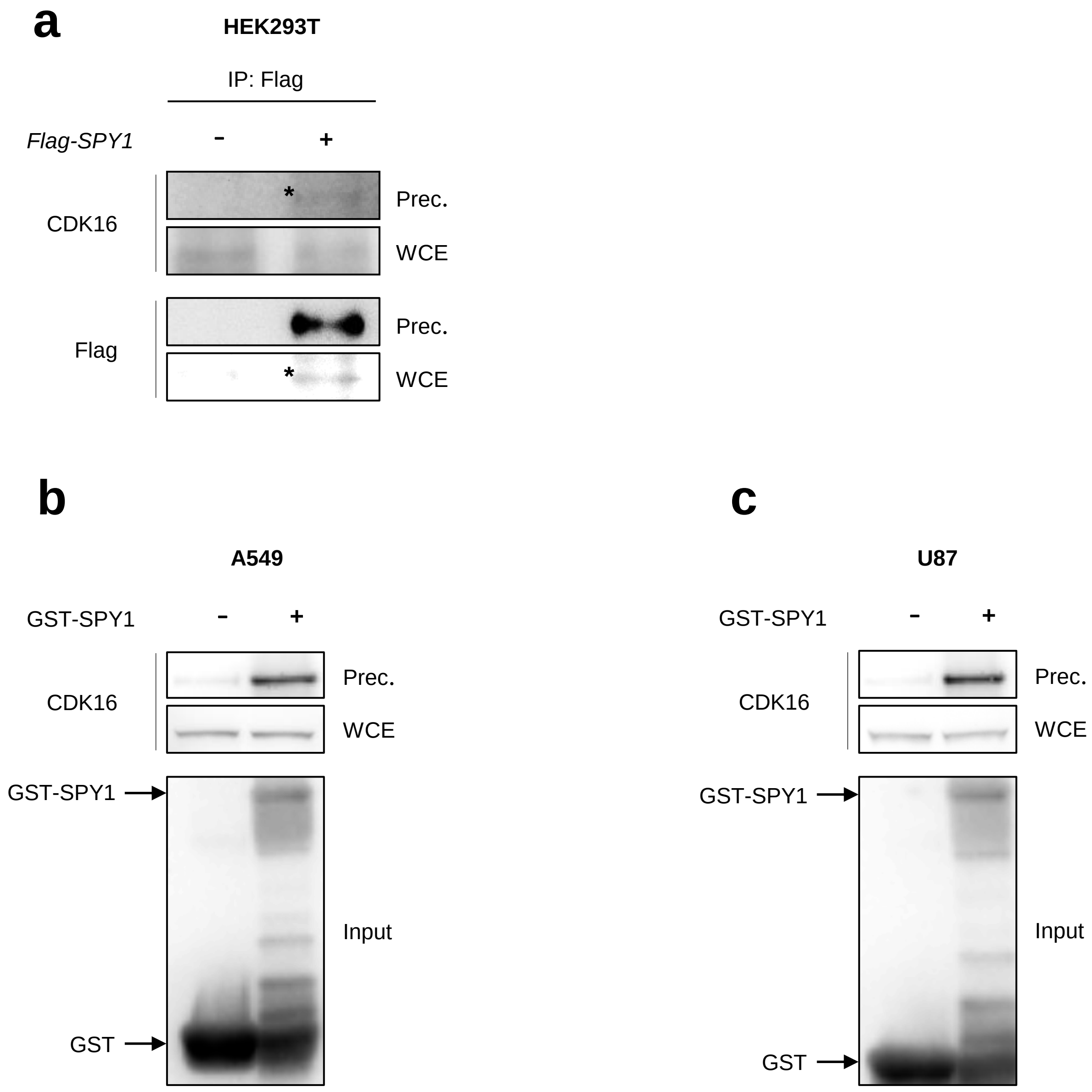

Supplementary Figure 2. Validation of the CDK16-SPY1 interaction. (A) HEK293T cells were transfected with empty pIRES2*eGFP* vector or with pIRES2*eGFP*-Flag-*SPY1*. Two days later, immunoprecipitation was performed using an anti-Flag antibody and the interaction was detected using an anti-CDK16 antibody. The whole cell extract (WCE) represents 0.6% of the protein amount used for the immunoprecipitation. (B, C) Extracts from A549 (B) and U87 (C) cells were incubated with GST or GST-SPY1 purified from *E. coli*. Endogenous CDK16 was detected using an anti-CDK16 antibody, while recombinant SPY1 was detected using an anti-GST. WCE represents 1.5% of the protein amount used for the trapping.

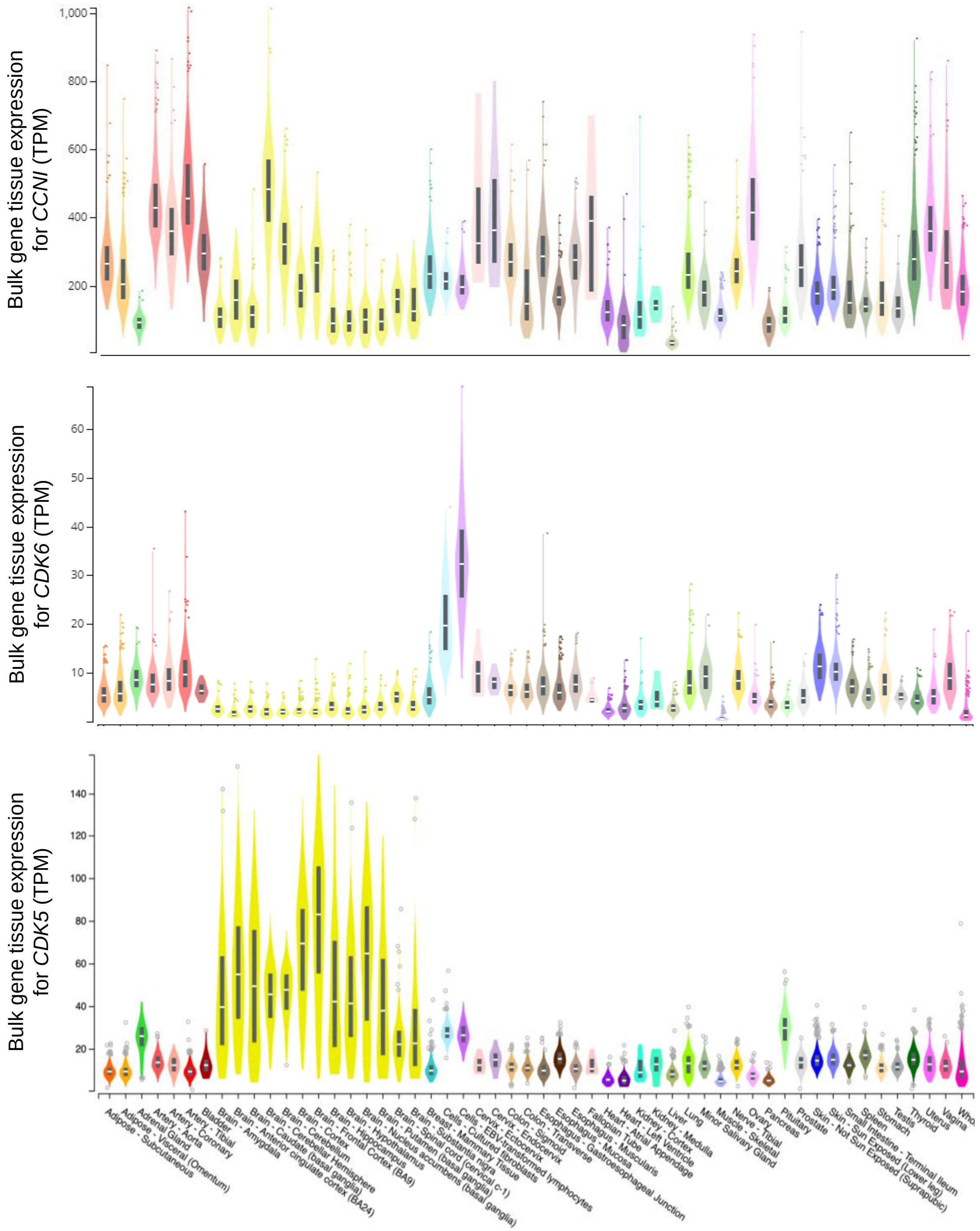

Supplementary Figure 3. ***CCNI*****,** ***CDK6*** **and** ***CDK5*** **expression in different tissues according to the GTEXPortal** (https://www.gtexportal.org/home/).

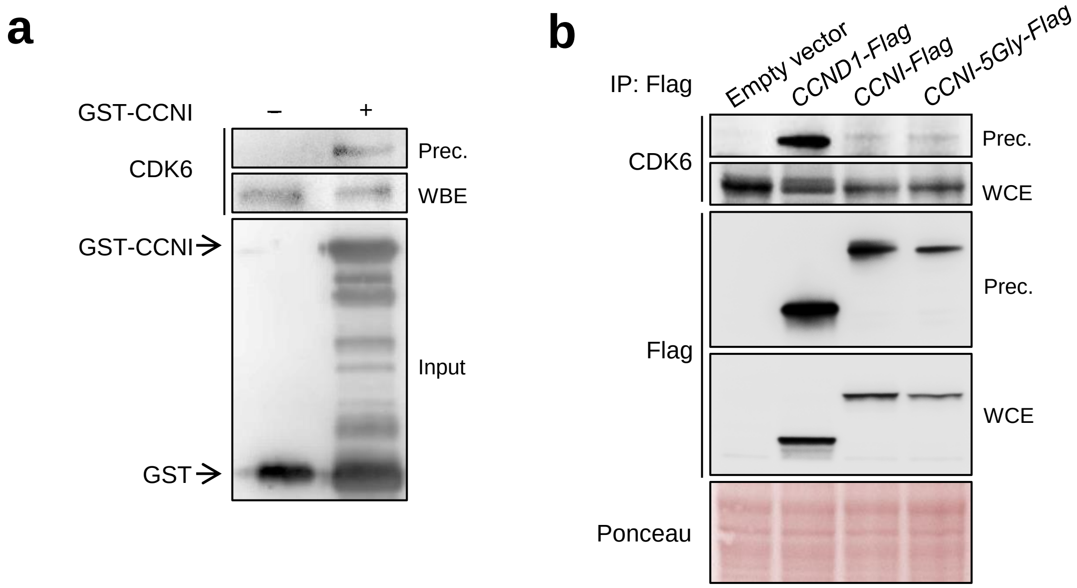

Supplementary Figure 4. Trapping and immunoprecipitation assays. (A) Trapping assay. Rat whole brain extract (WBE) was incubated with GST or with GST-CCNI purified from *E. coli*. CDK6 was detected by western blot using a specific antibody, whereas recombinant proteins were detected using an anti-GST. The image is representative of 2 independent experiments. (B) Immunoprecipitation assay. A549 cells were transduced with empty lentiviral vector or with the indicated Flag-tagged cyclins, which were immunoprecipitated using an anti-Flag antibody.

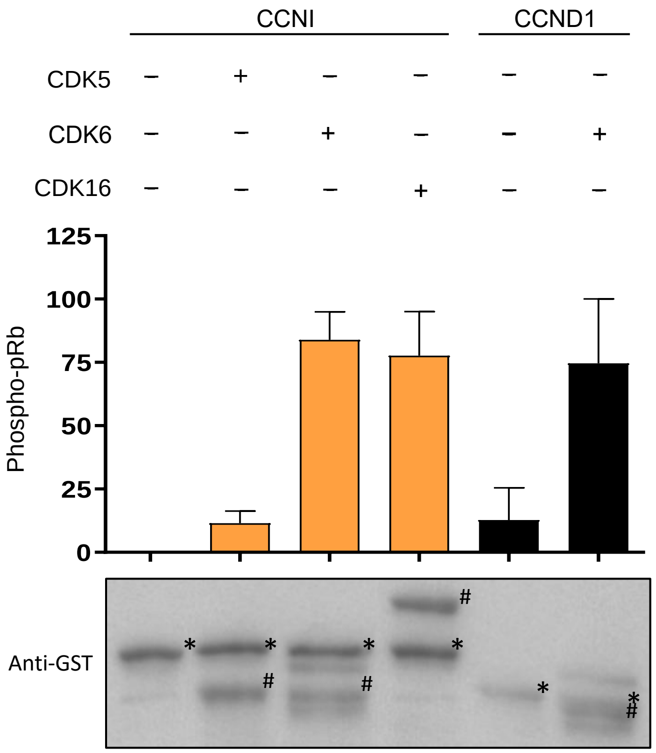

Supplementary Figure 5. Quantitation of the in vitro kinase assay shown in Figure 1E using pRb as substrate. Columns represent the mean ± SEM of at least 2 independent experiments. Anti-GST indicates the amount of cyclins (*) and CDKs (#) used to carry out the assay. Data was normalized using anti-GST signal intensity.

a

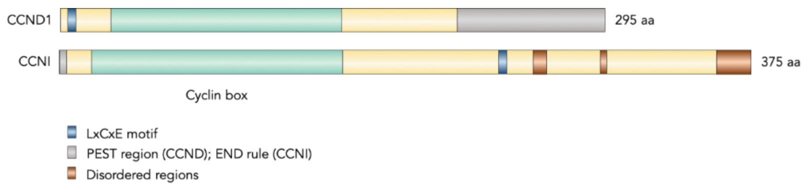

b

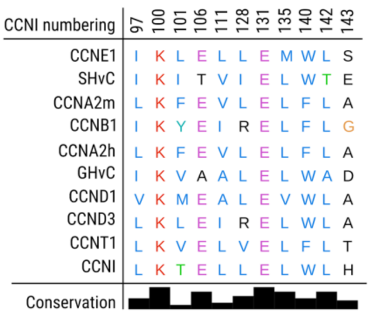

c

CCNI residue	Interface energy contribution (%)
TRP140*	7.45
GLU103	7.19
ASP141	4.83
LYS100*	4.76
PHE14	4.51
GLU104	3.86
ARG132	3.27
GLU131*	2.60
LEU128*	1.85
GLU64	1.85
SER124	1.83
THR101*	1.34
LEU135*	1.09
LEU142*	0.90
HIS143*	0.80
LEU97*	0.65

d

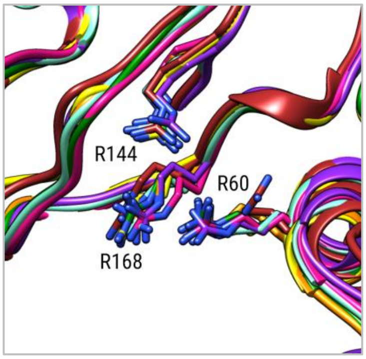

e

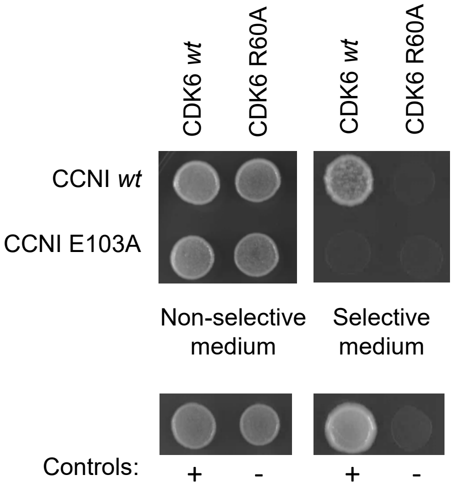

Supplementary Figure 6. Structural analysis of CCNI. (A) CCNI and CCND1 sequence comparison. The different linear motifs were detected using the ELM resource (http://elm.eu.org/). The length of the predicted Cyclin Box and disordered regions of CCNI were predicted with the Hammer resource (https://www.ebi.ac.uk/Tools/hmmer/) (Potter et al., 2018). The destabilization regions (PEST in the case of CCND1 or End-rule in the case of CCNI) were detected with the epestfind software (http://emboss.bioinformatics.nl/cgi-bin/emboss/epestfind). (B) Comparison of residue identities at the common interface positions among all CDK/Cyclin complexes. Numberings refer to the positions of the sequences deposited in the Uniprot database for CCNI (entry: Q14094) and CDK6 (entry Q00534). CCNE1: Cyclin E1, SHvC: Saimiriine herpesvirus 2 viral cyclin, CCNA2m: Mus musculus cyclin A2, CCNB1: Cyclin B1, CCNA2h: Homo sapiens cyclin A2, GHvC: Gamma herpesvirus cyclin, CCNT1: Cyclin T1. (C) Per-residue energy contribution to the total interface energy of the CCNI-CDK6 complex, expressed as the percentage of the residue interface energy to the total interface energy of the complex. Residues marked with an asterisk belong to the set of common interface positions in the cyclin/CDK complex. (D) Coalescence of the three arginines in several deposited structures (1JOW: violet, 1UNG: brown, 1XO2: green, 2EUF: aquamarine, 4TTH: yellow and 5L2W : yellow). Numberings refer to the sequences deposited in the Uniprot database for CCNI. (E) The interaction between CCNI E103 and CDK6 R60 was monitored by the yeast two-hybrid screening. Representative images of 2 independent experiments are shown. AgT-p53 was used as positive control, whereas AgT-Lam was used as negative control.

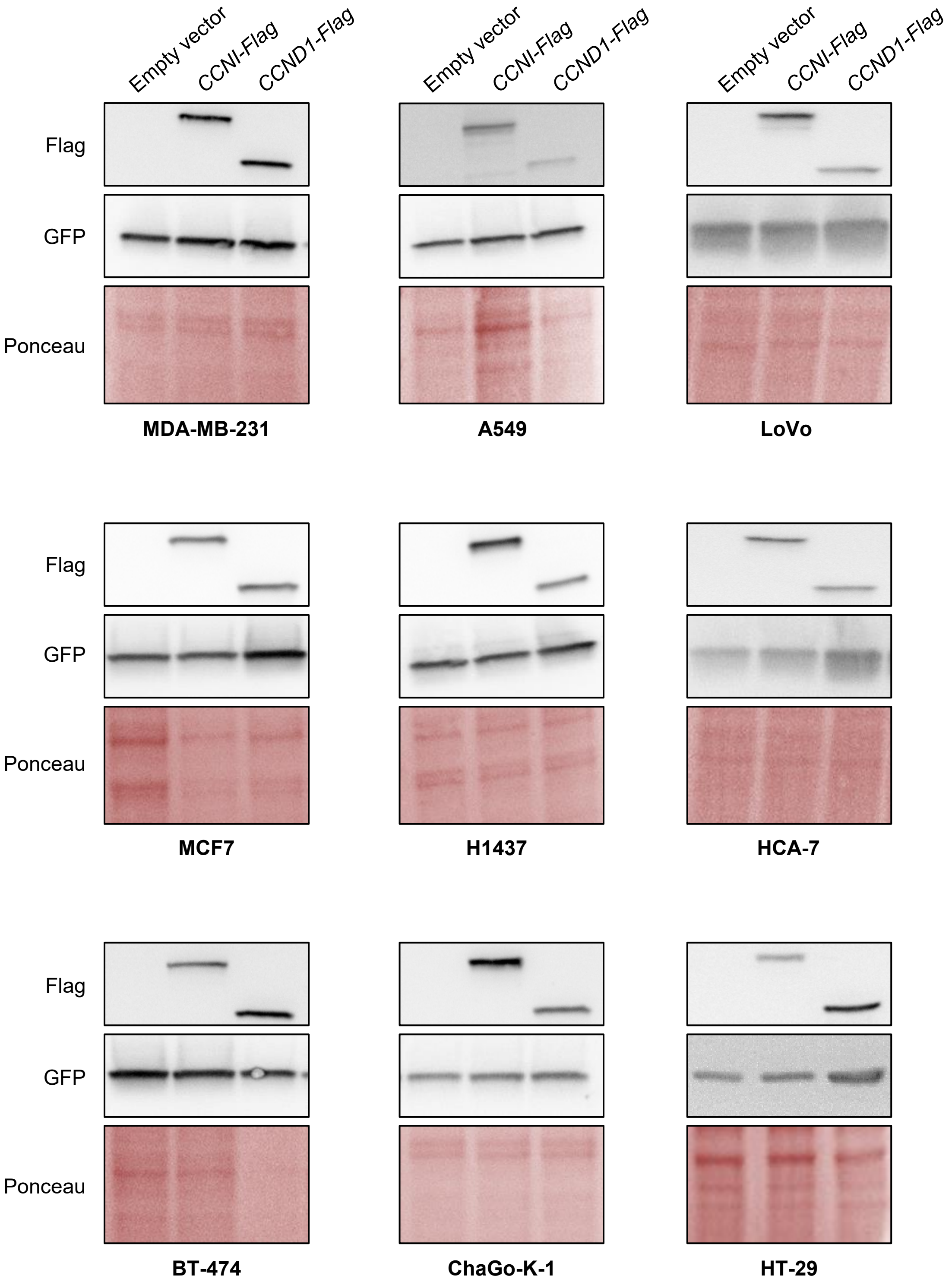

Supplementary Figure 7. Overexpression of *CCNI* and *CCND1* in cancer cells. Cells were infected with empty lentiviral vector (control) or with the indicated cyclin-overexpressing construct. Western blot analysis confirmed cell infection. Flag-tagged cyclins were detected with an anti-flag antibody and GFP was used as a reporter for lentivirus infection.

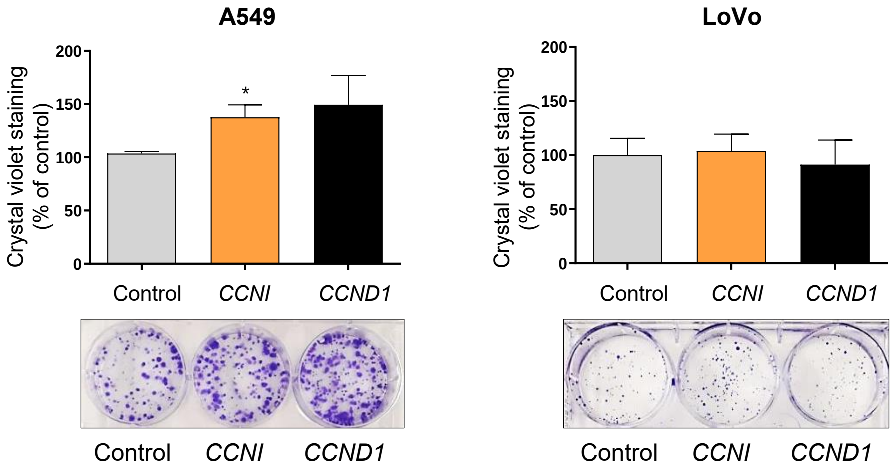

Supplementary Figure 8. Colony formation assays in A549 and Lovo cells. Cells were transduced with empty lentiviral vector (control) or with the indicated cyclin-overexpressing construct and the efficiency of cell colony formation was monitored 2 weeks later. Columns represent the mean ± SEM of 4 independent experiments performed in duplicates. Image below the graph shows representative images of colony formation assays. *P < 0.05 vs control, Mann-Whitney test.

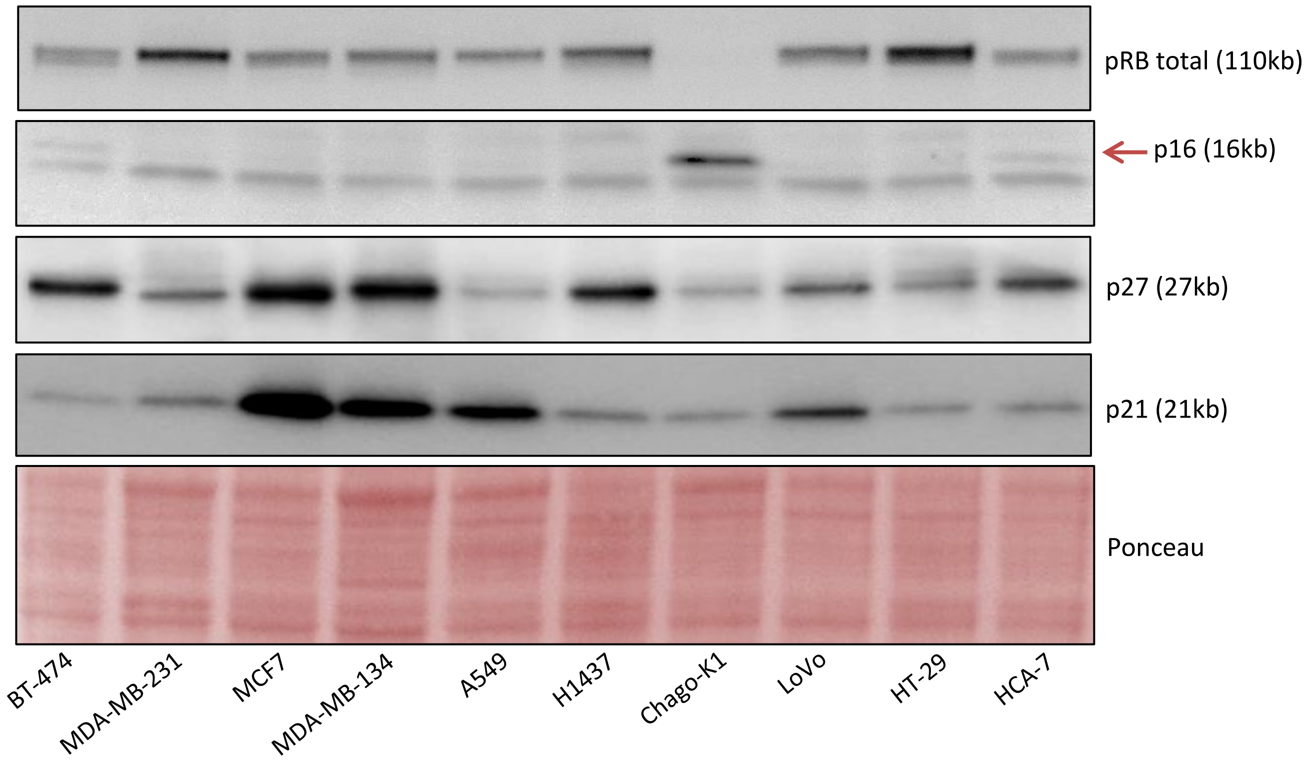

Supplementary Figure 9. Expression of cell cycle regulators in the cell lines displayed in Figure 2A. The protein expression of different cell cycle regulators in was monitored by Western Blot analysis.

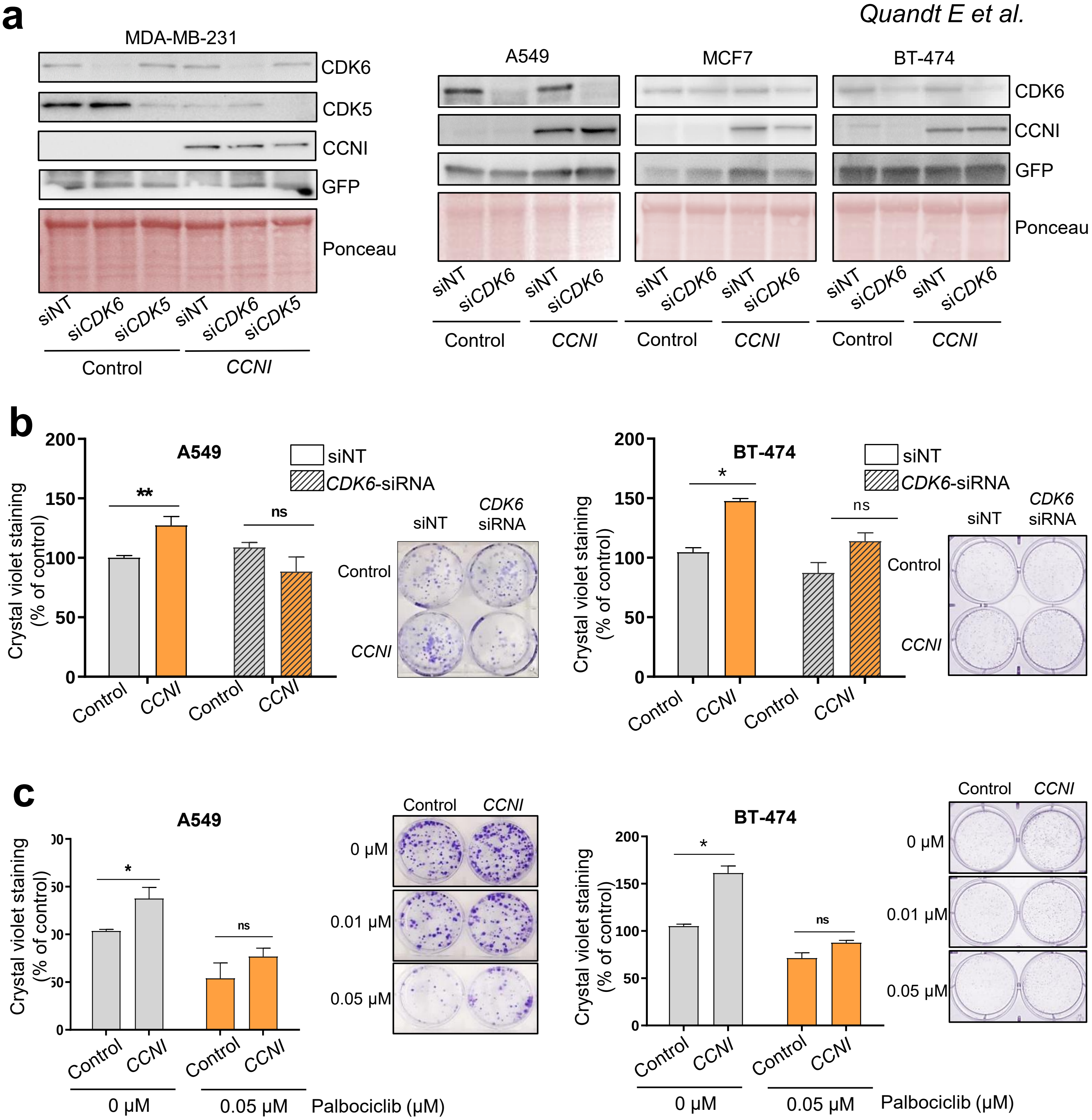

Supplementary Figure 10. *CDK6* silencing or inhibition abrogates CCNI-induced cancer cell proliferation. (A) Representative images of western blot analysis to confirm *CCNI* overexpression and *CDK6* or *CDK5* silencing. (B) A549 or BT-474 cells were infected with empty vector (control) or with the lentiviral vector expressing *CCNI*. Cells were then transfected with non-targeting siRNA (siNT) or with *CDK6*-targeting siRNA and, 48 h later, cells were seeded to assess colony formation 2 weeks later. Columns represent the mean ± SEM of 3 independent experiments performed in duplicates. **P < 0.01 vs the indicated control, Mann-Whitney test. Ns, not significant. (C) A549 or BT-474 cells were infected with empty vector (control) or with the lentiviral vector expressing *CCNI*. Cells were then seeded in 6-well plates and treated with the indicated concentrations of palbociclib. Colony formation was monitored 2 weeks later. Columns represent the mean ± SEM of 4 experiments performed in duplicates. *P < 0.05 vs the indicated control, Mann-Whitney test. Ns, not significant.

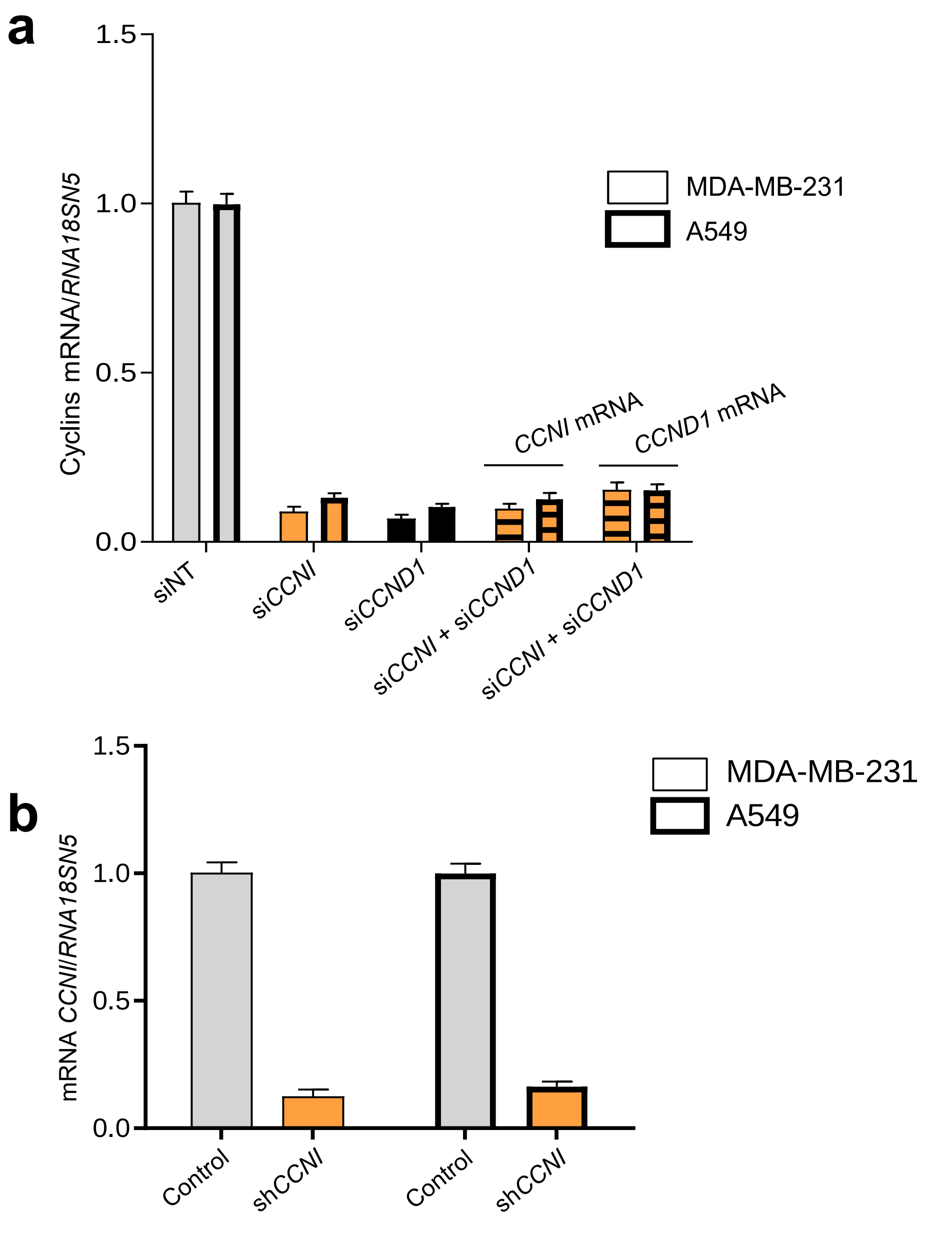

Supplementary Figure 11. Control of cyclins downregulation. (A) qPCR was performed to assess cyclin knockdown in figures 4A. The obtained values were normalized relative to the housekeeping gene *RNA18SN5* and calculated according to the $2^{-\Delta\Delta Ct}$ method. Columns represent the mean ± SEM of six (MDA-MB-231) or five (A549) independent experiments. (B) qPCR was performed to assess cyclin knockdown in figures 4B. The obtained values were normalized relative to the housekeeping gene 18S and calculated according to the $2^{-\Delta\Delta Ct}$ method. Columns represent the mean ± SEM of 4 experiments performed in duplicates.

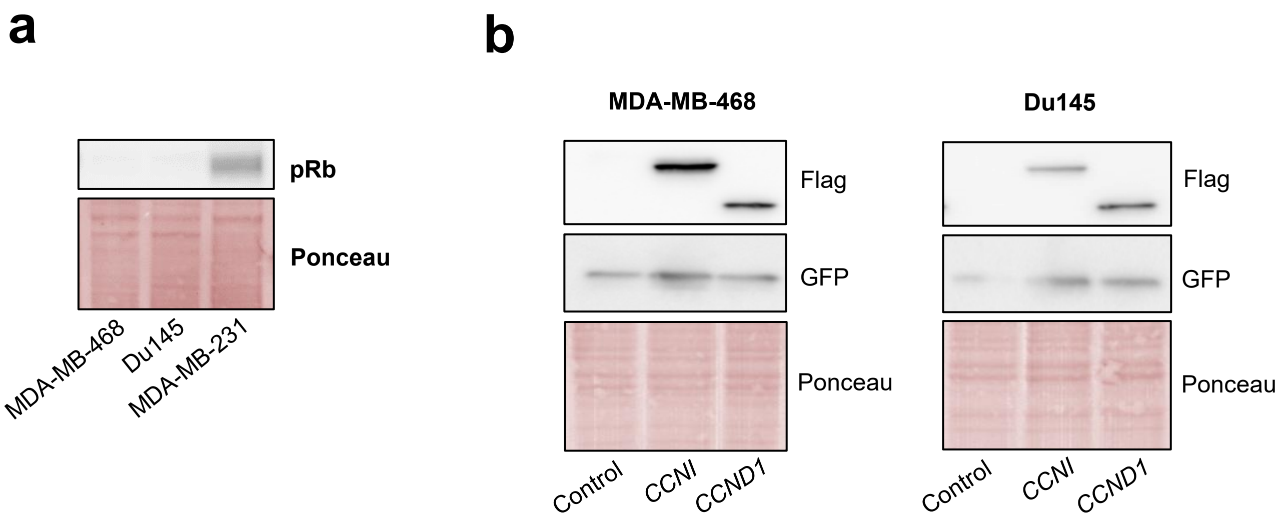

Supplementary Figure 12. *CCNI* overexpression in pRb-null cell lines. (A) pRb expression in the cancer cell lines displayed in Figure 4C. MDA-MB-231 cells were used as positive control. (B) Cancer cells were transduced with empty vector (control) or with the lentiviral vector expressing *CCNI* or *CCND1*. Western blot analysis confirmed cell infection. Flag-tagged cyclins were detected with an anti-flag antibody and GFP was used as a reporter for lentivirus infection.

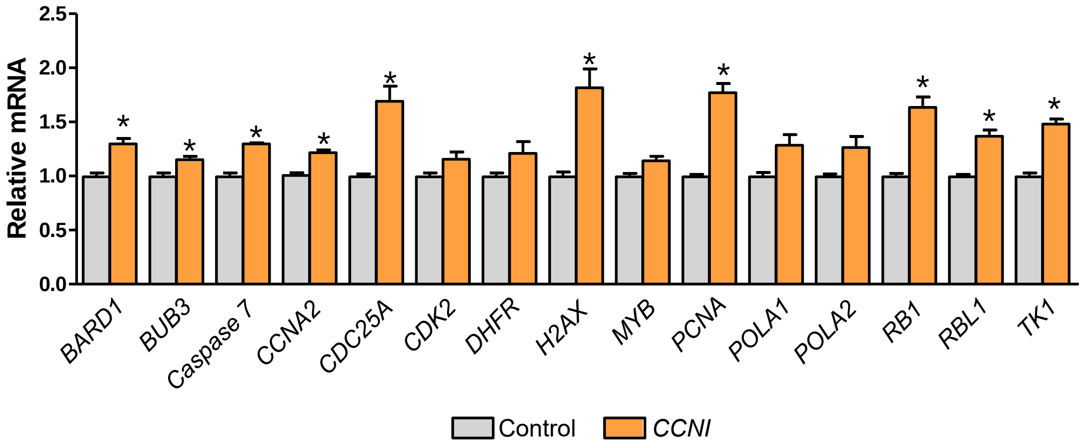

Supplementary Figure 13. Effect of *CCNI* upregulation on the expression of E2F target genes. MDA-MB-231 cells were transduced with empty (control) or *CCNI*-expressing construct and gene expression was monitored by RT-qPCR. The obtained values were normalized relative to the housekeeping gene *RNA18SN5*. Columns represent the mean ± SEM of 4 experiments performed in duplicates.

a

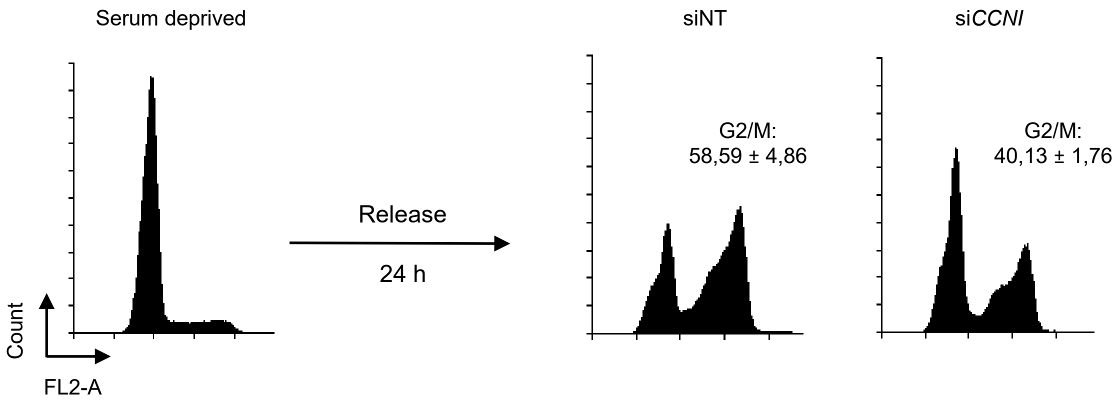

b

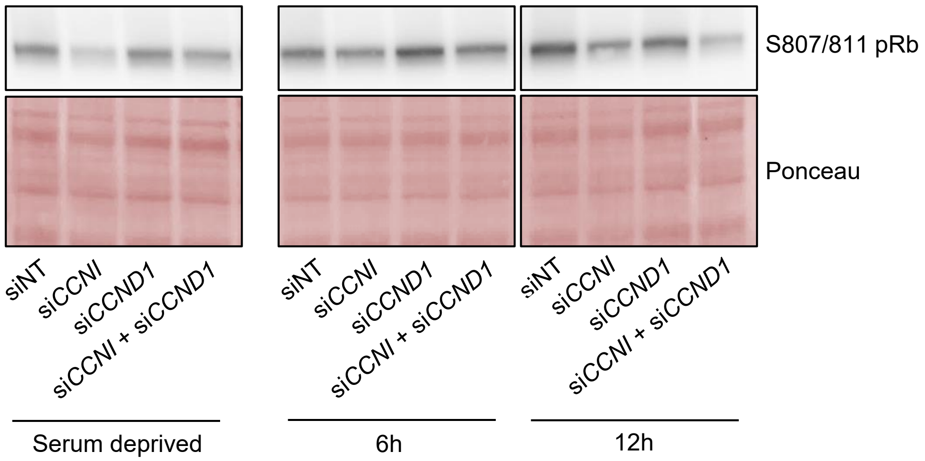

Supplementary Figure 14. Effect of *CCNI* downregulation on cell cycle entry. (A) A549 cells were seeded and transfected the following day with non-targeting siRNA (siNT) or with *CCNI* siRNA. 72 h later, cells were synchronized in G0/G1 by serum starvation and released into media containing 10% FBS, and nocodazole. Cells were collected for FACS analysis 24 h later. FACS profiles are representative of at least 2 independent experiments. Percentages indicate the mean ± SEM of at least 2 independent experiments. (B) A549 cells were transfected and synchronized in G0/G1 by serum starvation as described in (A). Cells were collected for western blot analysis at the indicated time points after release into medium containing 10% FBS. Images are representative of 2 independent experiments.

Supplementary Figure 15
Quandt E et al.

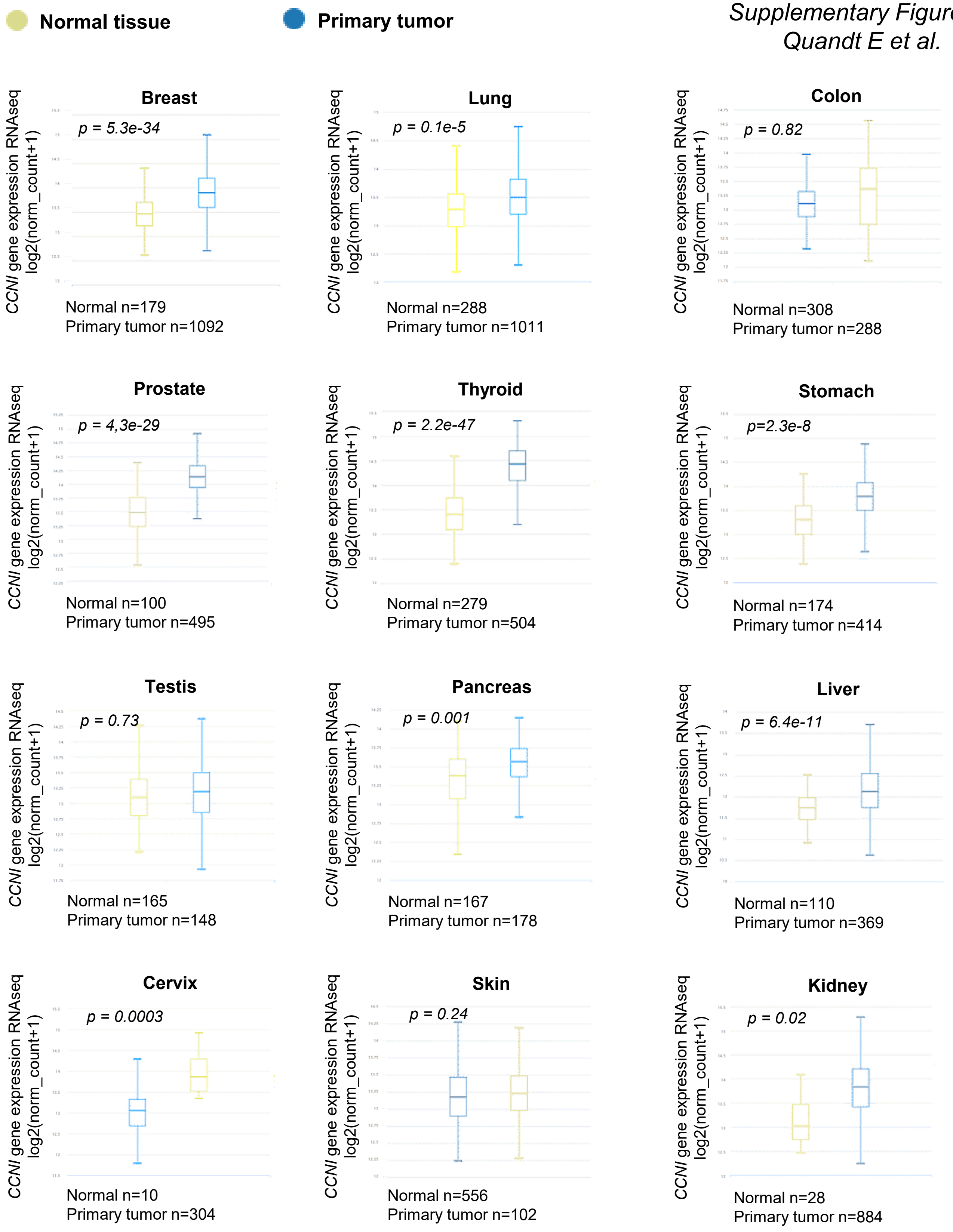

Supplementary Figure 15. *CCNI* expression in normal and primary tumor tissues. *CCNI* expression in normal tissues (shown in green) and primary tumors (shown in blue) according to GTEx and TCGA datasets, respectively, Welch's t-test. Data were obtained from UCSC Xena (https://xenabrowser.net/) on March 17th 2021.

Supplementary Figure 16
Quandt E et al.

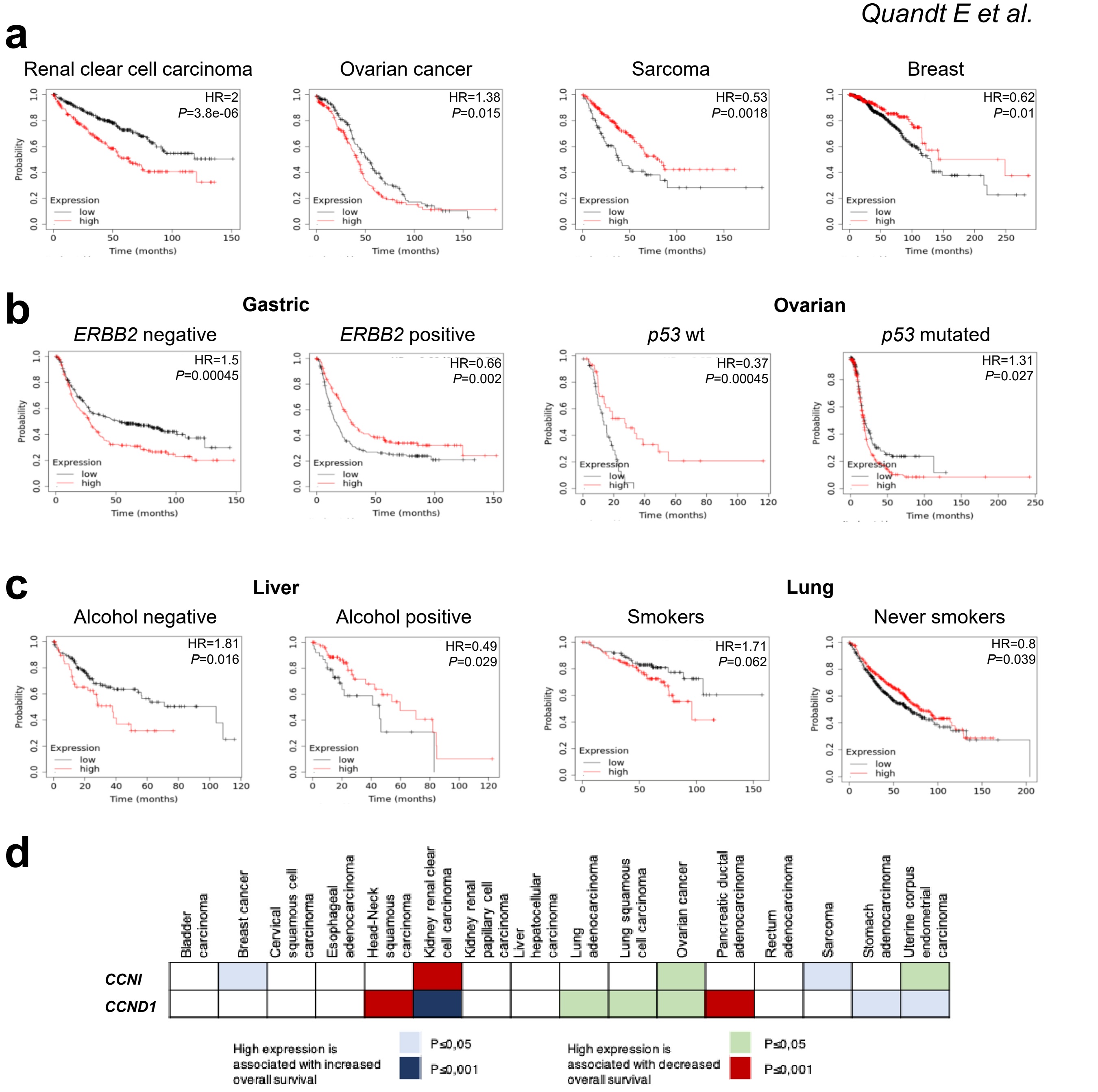

Supplementary Figure 16. The prognostic significance of *CCNI* depends on the disease context. (A-C) The overall survival of cancer patients exhibiting high (red) and low (black) expression of *CCNI* is represented using the Kaplan-Meier plotter software (http://kmplot.com/). (A) The prognostic value of *CCNI* is dependent on the tumor type. The 4 tumor types with a p-value < 0.05 in the Pan-Cancer (RNAseq) dataset are shown. (B) The prognostic value of *CCNI* is dependent on the mutation context. The tumor types where *CCNI* displays opposing effects in the Gene Chip dataset as a function of genomic background (*ERBB2* in gastric and *p53* in ovarian cancers) are shown. (C) The prognostic significance of *CCNI* depends on the clinical history. *CCNI* displays opposing effects as a function of alcohol consumption in liver (RNAseq) and smoking in lung (Gene Chip) cancer patients. (D) The image was built using the Kaplan-Meier plotter software and shows the overall survival of patients included in the Pan-Cancer RNA-seq dataset. Colors indicate significant association with a better or worse prognosis and statistical significance, as indicated in the figure key. Data were obtained on May 6th 2020.

Cat. No	Antibody	Species	Company
sc-6247	CDK5	Mouse	Santa Cruz Biotechnology
13331	CDK6	Rabbit	Cell Signaling
sc-7961	CDK6	Mouse	Santa Cruz Biotechnology
10102-1-AP	CDK16	Rabbit	Proteintech
F3165	Flag	Mouse	Sigma-Aldrich
A9521	G6PDH	Rabbit	Sigma-Aldrich
sc-577	Gal4	Rabbit	Santa Cruz Biotechnology
2956	GFP	Rabbit	Cell Signaling
CSB-MA000031M0M	GST	Mouse	CusAb
sc-805	HA	Rabbit	Santa Cruz Biotechnology
554136	pRb	Mouse	BD Pharmingen
9307	phospho-pRb (Ser780)	Rabbit	Cell Signaling
9308	phospho-pRb (Ser807/811)	Rabbit	Cell Signaling
80772	p16	Rabbit	Cell Signaling
2947	p21	Rabbit	Cell Signaling
sc-528	p27	Rabbit	Santa Cruz Biotechnology
ab92570	Thiophosphate ester	Rabbit	Abcam

Supplementary Table 1. List of antibodies used.

Gene (human)	Forward	Reverse
BARD1	5'-CATGCGAGACCCGATTCTGA-3’	5'-TAAACCAGCTCGAAGGAGCC-3’
BUB3	5'-GTGGGACTTACGGAACATGG-3’	5'-CACTCGGCCTTCAATAGAGC-3’
Caspase 7	5'-ACTGCTCTTGTGCCAAGATG-3’	5'-CATGGCTTAAGAGGATGCAG-3’
CCNA2	5'-TACCTGGACCCAGAAAACCA-3’	5'-CACTCACTGGCTTTTCATCTTCT-3’
CDC25A	5'-CACCAACCTGACCGTCACTA-3’	5'-GTTCTTCACCTCCAGTGGTTG-3’
CDK2	5'-TCCTCCACCGAGACCTTAAA-3’	5'-TACCACAGGGTCACCACCTC-3’
CTCF	5'-TTACACGTGTCCACGGCGTTC-3’	5'-GCTTGTATGTGTCCCTGCTGGCA-3'
DCK	5'-CCACCCCGCCCAAGAG-3’	5'- CTTCCCTGCAGCGATGTTCCC-3’
DHFR	5'-ATCGGCAAGAACGGGGA-3’	5'-TCTGGAAAGAAAATGAGC-3’
H2AX	5'-CGGGCGTCTGTTCTAGTGTT-3’	5'-GGTGTACACGGCCCACTG-3’
MYB	5'-GAAGGTCGAACAGGAAGGTTATCT-3’	5'-GTAACGCTACAGGGTATGGAACA-3’
PAICS	5'-GTCTTCTCTTCGACTACCCAGTGG-3’	5'-CAAATTGAGCTGATCCTTCTGGA-3’
PCNA	5'-TCCTCCTTCCCGCCTGCCTGTAGC-3’	5'-CGCGTTATCTTCGGCCCTTAGTGTA-3’
POLA1	5'-GCCAGGATGATGACTGGATT-3’	5'-TTGTTCGGTTTTGTCACTGC-3’
POLA2	5'-GACTATGAGTCGTTCTATGTTTACGC-3’	5'-ACAGACACAGCCGAGGACAT-3’
RAD21	5'-AGCGTGATGTTATCGATGAGCC-3’	5'-TTAACTCCCTGAGGTGGTGGTG-3’
RB1	5'-GAACATCGAATCATGGAATCCCT-3’	5'-AGAGGACAAGCAGATTCAAGGTGAT-3’
RBL1	5'-GCAACTACAGCCTAGAGGGAG-3’	5'-TGATACCCTTTCCAACCGTGG-3’
SMC3	5'- CAGCAAACTGAGGTACAATCCCA-3’	5'-CCGATGGCTGACTTGGTCAC-3’
TK1	5'-AGAAGGAGGTCGAGGTGATT-3’	5'-CACTGGGCAGTTCTCTTTGT-3’
TMPO	5'-TGCTCGCCTCCTGCCTGTAG-3’	5'-GACACAAAGCCAAGCCAGACC-3’
TRA2beta	5'-TCCAGAAGAAGCTCCCGAAG-3’	5'-TCTTGAACGCCTAGACTGCT-3’
USP1	5'-ACAGTCCTTAATCATTTCGGTTGA-3’	5'-GGAGTTGGCATGTTTCTTGAATGT-3’
RNA18SN5	5'-CTACCACATCCAAGGAAGGCA-3'	5'-TTTTTCGTCACTACCTCCCCG-3'

Supplementary Table 2. List of primers used.
